# The Color of Noise and Weak Stationarity at the NREM to REM Sleep Transition in Mild Cognitive Impaired Subjects

**DOI:** 10.3389/fpsyg.2018.01205

**Published:** 2018-07-17

**Authors:** Alejandra Rosales-Lagarde, Erika E. Rodriguez-Torres, Benjamín A. Itzá-Ortiz, Pedro Miramontes, Génesis Vázquez-Tagle, Julio C. Enciso-Alva, Valeria García-Muñoz, Lourdes Cubero-Rego, José E. Pineda-Sánchez, Claudia I. Martínez-Alcalá, Jose S. Lopez-Noguerola

**Affiliations:** ^1^Consejo Nacional de Ciencia y Tecnología, Mexico City, Mexico; ^2^Área Académica de Gerontología, San Agustín Tlaxiaca, Mexico; ^3^Centro de Investigación en Matemáticas, Mineral de la Reforma, Mexico; ^4^Facultad de Ciencias, Universidad Nacional Autónoma de México, Mexico City, Mexico; ^5^Universidad Nacional Autónoma de México, Querétaro, Mexico; ^6^Área Académica de Psicología, Universidad Autónoma del Estado de Hidalgo, San Agustín Tlaxiaca, Mexico; ^7^Division of Molecular Psychiatry, Department of Psychiatry and Psychotherapy, University of Medicine, Goettingen, Germany

**Keywords:** NREM to REM sleep, DFA, mDFA, stationarity, mild cognitive impairment

## Abstract

In Older Adults (OAs), Electroencephalogram (EEG) slowing in frontal lobes and a diminished muscle atonia during Rapid Eye Movement sleep (REM) have each been effective tracers of Mild Cognitive Impairment (MCI), but this relationship remains to be explored by non-linear analysis. Likewise, data provided by EEG, EMG (Electromyogram) and EOG (Electrooculogram)—the three required sleep indicators—during the transition from REM to Non-REM (NREM) sleep have not been related jointly to MCI. Therefore, the main aim of the study was to explore, with results for Detrended Fluctuation Analysis (DFA) and multichannel DFA (mDFA), the Color of Noise (CN) at the NREM to REM transition in OAs with MCI vs. subjects with good performances. The comparisons for the transition from NREM to REM were made for each group at each cerebral area, taking bilateral derivations to evaluate interhemispheric coupling and anteroposterior and posterior networks. In addition, stationarity analysis was carried out to explore if the three markers distinguished between the groups. Neuropsi and the Mini-Mental State Examination (MMSE) were administered, as well as other geriatric tests. One night polysomnography was applied to 6 OAs with MCI (68.1 ± 3) and to 7 subjects without it (CTRL) (64.5 ± 9), and pre-REM and REM epochs were analyzed for each subject. Lower scores for attention, memory and executive funcions and a greater index of arousals during sleep were found for the MCI group. Results confirmed that EOGs constituted significant markers of MCI, increasing the CN for the MCI group in REM sleep. The CN of the EEG from the pre-REM to REM was higher for the MCI group vs. the opposite for the CTRL group at frontotemporal areas. Frontopolar interhemispheric scaling values also followed this trend as well as right anteroposterior networks. EMG Hurst values for both groups were lower than those for EEG and EOG. Stationarity analyses showed differences between stages in frontal areas and right and left EOGs for both groups. These results may demonstrate the breakdown of fractality of areas especially involved in executive functioning and the way weak stationarity analyses may help to distinguish between sleep stages in OAs.

## 1. Introduction

### 1.1. Rapid eye movement sleep and mild cognitive impairment

**Figure 1:**
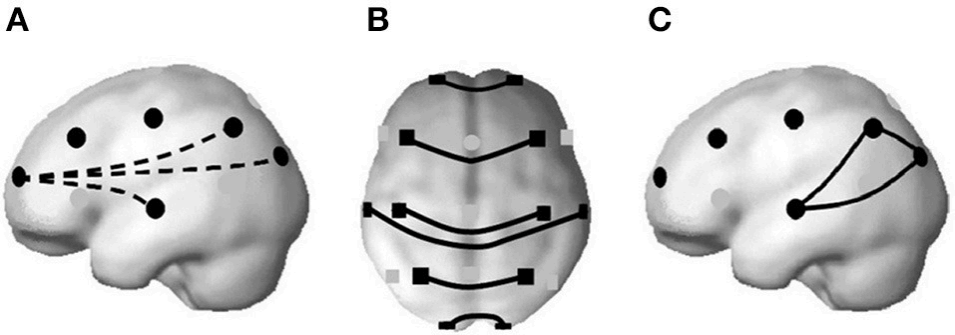
A decrease in temporal synchronization between frontal and posterior regions at higher frequencies has been found during REM sleep **(A)**. Instead, interhemispheric coupling increases at both anterior and posterior regions **(B)**; and also among posterior regions **(C)**. From Corsi-Cabrera (2018), based on the brain model of Okamoto et al. (2004), with permission.

Rapid Eye Movement (REM) sleep enhances the information flow among functional networks. Well-defined cortico-cortical and thalamo-cortical networks (Steriade and Amzica, 1996) operating at higher frequencies that contrast with the great synchronizations in Slow Wave Sleep (SWS) have been demonstrated (Steriade et al., 1996). During REM sleep, in comparison to the wakeful state and Non-Rapid Eye Movement (NREM) sleep in humans, a decoupling of frontal vs. posterior areas occurs (Pérez-Garci et al., 2001; Corsi-Cabrera et al., 2003), and temporal coupling increases among homologous regions of the two cerebral hemispheres and among posterior regions (Corsi-Cabrera et al., 1987, 1989), as can be represented in Figure 1.

Evidence supporting these investigations has been found involving selective REM sleep deprivation effects over the interhemispheric correlation in gamma frequencies on the recovery night in frontal lobes (Corsi-Cabrera et al., 2014) and a higher prefrontal rebound of frontal gamma synchronization during subsequent wakefulness in young subjects performing executive tasks (Corsi-Cabrera et al., 2015).

Language, planning, purposive action, voluntary control of attention, working memory, evaluation, decision making, inhibition of stimuli and irrelevant responses, sequential organization of new and complex information (Fuster, 1999), as well as rule guided behavior have been together classified as executive functions (Fuster, 2005; Bunge and Wallis, 2008). In Mild Cognitive Impairment (MCI) it has been widely demonstrated that some of these functions are distorted. Patients diagnosed with MCI may have subjective mental complaints about their cognitive functioning, corroborated by a near relative or friend, or lower performances considering age and education standards on neuropsychological tests (Petersen, 2004).

Physiological indicators of cognitive impairment in the elderly during REM sleep comprise greater absolute and relative power in slower frequencies in frontal lateral regions vs. wakefulness (Brayet et al., 2015) and less muscle atonia (Chen et al., 2011). The above mentioned studies have included linear analyses of the Electroencephalogram (EEG) or the Electromyogram (EMG). In this paper, Detrended Fluctuation Analysis (DFA) and multichannel DFA (mDFA) are used to calculate the level of fractality in the NREM to REM transition from subjects with and without cognitive impairment.

REM sleep has long been associated with cognitive functions (Rasch and Born, 2013; Tononi and Cirelli, 2014). REM sleep plays a role in memory consolidation (Boyce et al., 2016, 2017; Peever and Fuller, 2016). After complex tasks, there are reactivations of neural circuits during REM sleep (Louie and Wilson, 2001). Long Term Potentiation (LTP) only happens during wakefulness and REM sleep, and, depending on its theta phase of the hippocampus, LTP can be enhanced or inhibited (Pavlides et al., 1988).

Generally, three REM sleep indicators appear in consecutive order. When entering this stage, spindles and high amplitude slow waves are absent, the EEG has abundant beta and gamma frequencies (Llinás and Ribary, 1993; Steriade and Amzica, 1996), there is an abrupt loss of voltage that occurs at an interval shorter than 2 seconds (Rosales-Lagarde et al., 2009), and, afterwards, the characteristic episodic REMs appear (Aserinsky and Kleitman, 1953; Rechtshaffen and Kales, 1968; AASM, 2007).

Evidence suggests the need to search for indicators of cognitive impairment in REMs. Before REMs, an internal attentive network comes into operation, for these are preceded by higher activations of the orbital region, amygdala and hippocampus (Ioannides et al., 2004) and a higher temporal coupling between the right frontal region and the midline (Corsi-Cabrera et al., 2008). Theta activity and Ponto-Geniculo-Occipital (PGOs) waves come into phase and their generators receive a common activation (Karashima et al., 2002).

It is proposed that, given the important role of REM sleep for cognitive functions, scaling exponents must differ for people with MCI vs. controls in at least one of the sleep markers.

To examine the functional relationships mentioned above, the analyses included individual derivations, bilateral, anteroposterior and posterior networks at the EEG. The Electrooculogram (EOG) was subjected to DFA and mDFA as explained below, and the scaling exponents of the EMG were also obtained. In brief, according to the fractal analysis of signals, it is known that if the noise of the signal is pink or toward 1, the signal represents health or a good correlation measure along a wide range of signals. On the contrary, in large scales, white noise means the signal is random; over short scales, brown noise is similar to the Brownian movement.

According to Nikulin and Brismar (2005), drowsy states have higher Hurst values than those of wakefulness. In Linkenkaer-Hansen et al. (2001) studies, the eyes-open condition -comprising beta frequencies- has smaller scaling values than the closed-eyes conditions, where alpha frequencies at parietal and occipital regions appear. In Weiss et al. (2009) and Weiss et al. (2011), healthy and younger subjects than ours were evaluated to obtain the Hurst values for the different sleep stages. To compute the Hurst Exponent, Weiss et al. (2009) and Weiss et al. (2011) employed the R/S statistics or rescaled adjusted range of Mandelbrot and Taqq (Mandelbrot, 1977). They found higher values in Hurst measures in NREM stage 4 versus NREM stage 2 and REM. Hurst values at REM sleep in frontal regions were lower than in all stages.

A difference between the signal of both groups is expected if the structural complexity of dendritic arborizations that possess a fractal anatomy is distorted in the group with impairments. Also, the replay of activity in sleep that may mimic the one during wakefulness and could be associated with the functional relationships, i.e., inverse functional relationship found by Corsi and collaborators mirrored by a decrease at the cross-correlations of anteroposterior (Pérez-Garci et al., 2001; Corsi-Cabrera et al., 2003) and an increase in posterior and interhemispheric networks (Corsi-Cabrera et al., 1987, 1989), may be altered in the MCI group. Although these results are based on linear analyses and the present paper employs DFA and mDFA, there are evidences supporting lost long range anatomical connections in patients with MCI and dementia (Andrews-Hanna et al., 2007). Especially, heteromodal association networks such as frontoparietal and hippocampus connectivity become more vulnerable with age and dementias than short range connections, such as sensoriomotor primary cortices connections (Li et al., 2017). Considering the results of Weiss et al. (2009) showing greater values in the SWS stages and the study of Brayet et al. (2015), revealing slower activity in MCI subjects may have greater Hurst values that would reveal their cognitive disadvantages.

Also, since in a previous report (Rosales-Lagarde et al., 2017) the percentage of stationarity of REM sleep was lower than that of NREM sleep and wakefulness, the degree of stationarity was obtained as an index to compare NREM vs. REM sleep in both groups.

## 2. Methods

### 2.1. Subjects

Most of the 115 Older Adults (OAs) evaluated with the cognitive and emotional tests mentioned below attended the Centro Gerontológico Integral (CGI) at Punta Azul in Pachuca, Hidalgo, Mexico. OAs were informed about the aims of the research and they signed an informed consent. The project received the approval of the research Ethics committee. A clinical interview was first applied to rule out epilepsy or psychiatric disorders. OAs were also asked about personal and family diseases. Cognitive assessment by the Neuropsi and the Mini-mental State Examination (MMSE), as well as the emotional evaluation by the Geriatric Depression Scale (GDS) and a Scale for the detection of Anxiety for the Elderly (the Short Anxiety Screening Test: SAST), were administered. The Katz Index of Independence in Activities of Daily Living (Katz et al., 1970) was also administered to rule out dementia, and all tests were validated in Spanish (Ugalde, 2010). The tests were rated by trained experts (ARL and GVT). Later, according to the results of the tests, the OAs were divided in two main groups, one with normal functioning on the Neuropsi and all its scales and subscales, with undiminished daily activities or control (CTRL), and another group with similar results in the daily living tests but with at least one subscale on the Neuropsi showing three standard deviations below the mean, or with MCI. Table 1 shows the results for 13 subjects, 6 for the MCI group and 7 for the CTRL group. Another subject who was registered and classified as having MCI had facial paralysis and, due to technical problems, did not complete the whole PSG study.

**Table 1:** Demographic and clinical characteristics by group.

	**CTRL**	**MCI**	***t***	***p***
**Demographic characteristics -M (SD)**				
Age	64.5 (9)	68.1 (3)	0.90	0.39
Education (years)	11 (5)	8.5 (2)	1.08	0.30
**Sex -n(%)**				
Male	1 (14.2)	4 (66.7)		
Female	6 (85.7)	2 (33.3)		
**Clinical assessment -M (SD)**				
MMSE	29.0 (1)	27.50 (1)	2.01	0.07
Katz	0.14 (0.3)	0.17 (0.4)	0.11	0.92
GDS	2.8 (3)	4.17 (3)	0.79	0.45
Sast	20.4 (4)	18.83 (4)	0.64	0.54
**Clinical characteristics -n (%)**				
Hypertension	3 (42.8)	4 (66.7)		
Diabetes	2 (28.5)	2 (33.3)		
Thyroid problem	1 (14.3)	0 (0)		
Sleep complaints SAST	2 (28.5)	2 (33.3)		
Memory complaints GDS	0 (0)	1 (16.6)		

*CTRL, Control group; MCI, Mild Cognitive Impairment; MMSE, Mini-Mental State Examination; GDS, Geriatric Depression Scale; SAST, Short Anxiety Screening Test; p < 0.05*.

As presented in Table 1, participants did not differ as regards age or education. The MMSE showed marginal differences between groups and daily living activities were maintained. Hypertension, diabetes and a thyroid problem were under medication. In answer to the question “Do you sleep well?” on the SAST scale of anxiety, 2 members of the MCI group and 2 of the CTRL group responded “never or rarely” or “occasionally.”

Memory complaints as measured on the GDS by the only question about referring to altered memory were scarce, because only one subject from the MCI responded affirmatively. If the question was about having problems to concentrate, the same subject from the MCI group answered positively. Nevertheless, when the question was about the mind being as clear as before, four CTRLs answered negatively and only two of the MCI group did, though objectively there were several scores below the mean in memory subscales in the MCI group.

### 2.2. Neuropsychological testing

The Neuropsi test measures neuropsychological functions. It was developed in the Universidad Nacional Autónoma de México and has been validated in Mexico with standards varying according to age and educational level (Ostrosky-Solís et al., 1999). The maximum score on the test is 130. Neuropsi test has clearly distinguished between normal, cognitive impaired and demented subjects (Ostrosky-Solís et al., 1999; Abrisqueta-Gomez et al., 2008; Montes-Rojas et al., 2012) and comprises several scales and subscales:

- Orientation in three dimensions: time, place and person.
- Attention and concentration: digits in reverse order, visual detection and subtraction.
- Memory: coding of three lists of words, coding of a visuospatial figure. Spontaneous evocation of the words and the figure. Evocation of the same words by categories and recognition of them.
- Language: identification of figures in drawings and repetition of words and a sentence. Comprehension of instructions about figures. Verbal fluency and semantic fluency.
- Reading and writing. In reading, a short text is read aloud and three questions are asked about it. In writing, a dictation takes place. Also, the subject must copy a sentence.
- Conceptual and motor executive functions. The former include: similarities between two words. Calculations and following a certain written sequence. Motor executive functions: repeating movements first with the right and then with the left hand, alternating hand movements and finally, reacting with an opposite hand movement.

However, we excluded one of the subscales from the analysis as a basis for diagnosing MCI because only one subject from the 13 subjects could correctly continue the written sequence. Significant differences in the total score of the Neuropsi and the scales of “Attention,” “Memory,” and “Executive functions” were found between the groups (Table 2).

**Table 2:** Mean and standard deviations of the groups and their comparisons for Neuropsi scores.

	**CTRL**	**MCI**	***t***	***p***
Total score	110.0 (5)	91.7 (10)	4.0	**0.001**
Orientation	6.0 (0)	5.8 (0.4)	1.0	0.30
Attention and concentration	22.5 (1)	17.3 (5)	2.4	**0.03**
Memory	38.0 (3)	30.0 (5)	3.3	**0.01**
Language	22.7(1)	21.3 (2)	1.6	0.14
Writing/reading	4.7 (1)	4.8 (0.4)	0.3	0.74
Executive functions	16.0 (1)	12.1 (1)	5.9	**0.001**

*CTRL, Control; MCI, Mild Cognitive Impairment. Student t-tests, p < 0.05. Significant results for Student t-tests are indicated in bold*.

### 2.3. Procedure

The administration of the battery of tests was carried out either at the CGI or at the Polyclinic belonging to the Department of Gerontology of the School of Health Sciences of the Universidad Autónoma del Estado de Hidalgo. OAs who satisfied the criteria were registered at the Laboratory of Sleep, Emotion and Cognition, under the direction of AR-L, located at the Polyclinic. OAs filled a sleep questionnaire and received instructions to continue their normal activities prior to the study and were told to avoid alcoholic drinks or energizers during 24 h before the study, and not to take naps the day they were to stay in the laboratory. OAs were scheduled to arrive in the afternoon, at least 4 h before their normal bedtime, and performed some cognitive tasks (not presented here) before going to sleep.

### 2.4. Polysomnography

The EEG was registered with a MEDICID-5 with 26 amplifiers. 19 silver chloride electrodes were located according to the International 10–20 System (FP1, FP2, F3, F4, F7, F8, C3, C4, T3, T4, T5, T6, P3, P4, O1, O2, FZ, CZ, and PZ) and linked-ears were used as reference. The EMG was measured bipolarly with two electrodes located on the chin. The EOG was registered monopolarly with 2 electrodes: one a centimeter above and one below the external edge of each eye and also referred to linked-ears. A leg electrode was also located to detect if Restless Leg Syndrome (RLS) was present. Due to technical problems, no account was retained of leg movements in 6 of the 13 subjects (one from the MCI group and five from the CTRL group), so no statistic analysis about periodic leg movements could be performed to compare the groups. Filters were set between 0.1–100 Hz for the EEG, 10–70 Hz for the EMG, and 0.3–15 Hz for the EOG. A notch filter was centered at 60 Hz to avoid contamination. Impedance was kept below 10 kΩ. Data were digitalized with a sample frequency of 512 Hz and an A/D convertor of 16 bits and were stored on the same MEDICID-5 system. The register began after calibrating the signals and bidding the participant good night. In the morning, OAs filled a Likert scale about their quality of sleep. LC-R, a trained Neurophysiologist, analyzed the signals offline to score the registered data. Sleep was classified by the AASM criteria (AASM, 2007) and the following sleep architecture variables were calculated: Total Time in Bed (TTB), Total Sleep Time (TST), sleep efficiency, latencies, number of minutes and percentages of sleep stages N1, N2, and N3, REM, Wakefulness After Sleep Onset (WASO), number of leg movements, index of periodic leg movements per hour of sleep, number of waking periods per night, index of waking periods per hour of sleep, number of arousals per hour of sleep and index of arousals per hour of sleep.

Signals were converted to the text format and DFA and mDFA were obtained with the MATLAB version R2015 (see data in Supplementary Material for the details of the custom code in MATLAB). As stated below, each 30 s epoch was tested for its degree of stationarity or non-stationarity with the free R statistical software.

### 2.5. Statistical analysis

#### 2.5.1. Cognitive tasks

Age, educational levels and total scores on each subscale of the Neuropsi and MMSE were compared between groups using independent Student *t*-tests. As presented in Table 1, age and educational levels did not show significant differences between groups, and to explore the relationship between age and educational level, a Pearson's correlation analysis was performed, revealing an almost perfect negative correlation. Also, a correlation analysis between the Neuropsi and the MMSE was carried out to verify the equivalence of Neuropsi and MMSE as tests to diagnose cognitive impairment. To rule out possible effects of age on the Neuropsi, another correlation test was made between the total score of the Neuropsi and age.

#### 2.5.2. Polysomnography

Ten epochs of 30 s mostly consecutive before the first episode of REM sleep and ten during REM sleep were scored for each subject. The DFA and the mDFA for each epoch and each group were calculated. Some subjects had not enough epochs at this episode and another REM episode was chosen. Epochs with artifacts were rejected by visual inspection. Rejection applied to few subjects only, affecting 0 and 46 epochs for the CTRL group in NREM and REM, respectively; and to 6 and 15 for the MCI group within NREM and REM sleep, respectively. Pearson's chi-square tests were performed for each stage to discard any unequal contribution of N1, N2, or N3 stages. Likewise, chi-square tests were performed for the comparison of the number of arousals and the index of arousals per hour of sleep. When the frequency was lower than 5, the Yates correction was used.

Correlation analyses were performed between the Neuropsi scores and the Hurst exponents considering the mean of the ten epochs for each stage at individual derivations and multichannel values; another correlation was done between the Hurst exponents of these stages and age. Kolmogorov-Smirnov tests were performed and all DFA and mDFA data followed normal distributions. Also, mixed ANOVAs (2 × 2) were used to test for statistical differences, with group (CTRL and MCI) as the between-subject factor and condition (NREM and REM) as the within-subject factor, using DFA and mDFA for each channel and assessing the interhemispheric relationship (bilateral channels including the EOG), anteroposterior (FP1-P3 and FP2-P4) and posterior networks (O1-P3-T3 and O2-P4-T4). First, only the means of the NREM and REM epochs were considered for the analysis. Next, another mixed ANOVA was performed not taking into account the means but instead the ten values for each set of channel and multichannel contrasts, using the Greenhouse-Geisser's correction for repeated measures (see Figure S1 in Supplementary Material to see the detailed figures for the NREM to REM transition). By visual inspection one subject from each group appeared to be an outlier, so these two subjects were removed and the ANOVAs were repeated. All subjects were kept because the differences were not qualitatively significant as can be shown at (see Table S2 in Supplementary Material). Wilcoxon tests were performed for stationarity percentages between the ten epochs of each group and stage and throughout the whole register (see Table S1 in Supplementary Material to see the detailed percentages for the groups). Bonferroni corrections for multiple comparisons between channels were not performed, because these criteria would eliminate our results and so the present findings must only suggest a tendency.

## 3. The color of noise

The power spectrum *P* of a time series is calculated using the Discrete Fourier transform. Many natural phenomena show a characteristic curve that can be fitted to the functional form:

(1) $$\begin{array}{r} P\left(f\right)\propto f^{\alpha } \end{array}$$

where *f* is the frequency. In this case, the power spectrum is said to scale as a *power law*. Power laws are abundant in nature, and a few examples include allometric laws in Biology (West et al., 1997), the inverse-square laws of Newton and Coulomb (Heering, 1992), Kepler's third law (Gingerich, 1975), etc. It is worth noticing the fact that the expression 1 implies *scale-invariance* (Song et al., 2005): multiplying *f* by a constant factor *a* gives *P*(*af*) = (*af*)^α^ = *a*^α^*P*(*f*) ∝ *P*(*f*). In other words, the curve *P*(*f*) is invariant to changes in the scale of the independent variable since the resulting plot is the same as the original but scaled by the factor *a*^α^ in the dependent variable.

**Figure 2:**
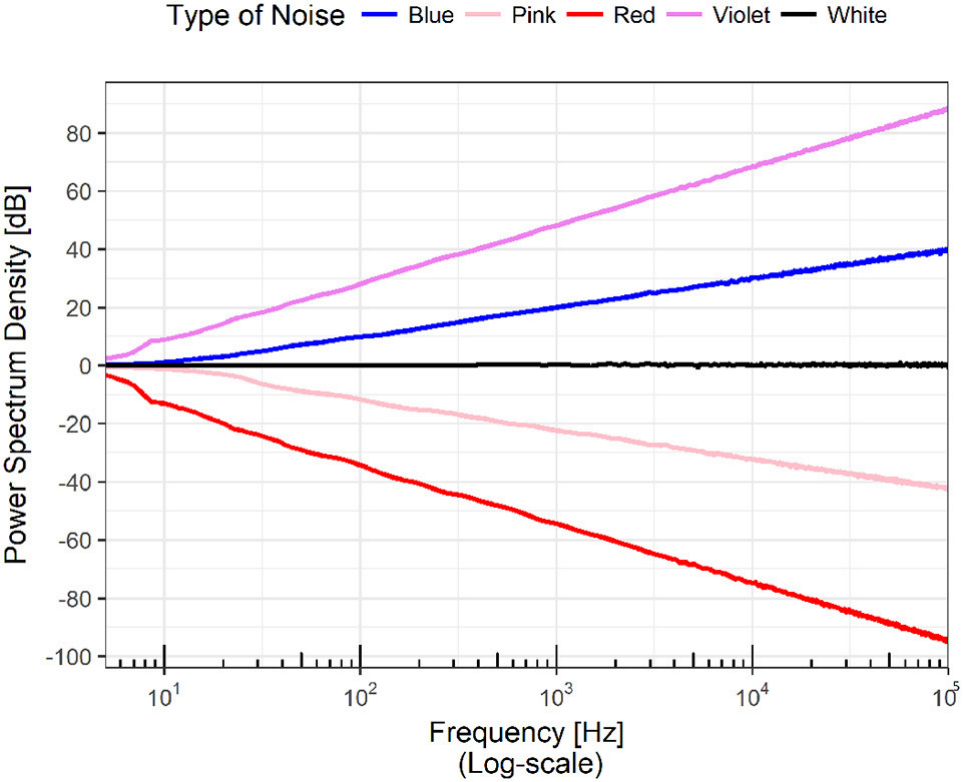
The figure shows the power spectra of a set of time series. The scale of both axes is logarithmic, meaning that the plots are power laws in linear scales; that is, a family of parabolas or hyperbolas depending on the sign of the exponent. A horizontal line in the power spectrum means that all the frequencies appear in the Fourier transform of the time series with the same power. In analogy with the visible electromagnetic radiation, this case is know as “white noise.” Of special interest are the cases when the slope of the line is -1 and -2. In the first scenario, the time series has all the frequencies but the low ones dominate and thus it has a mixture of white and red and the result is called “pink noise.” When the slope is -2, the resulting noise (it has all the frequencies) is called “Brown” not because the mixture of frequencies lead to that color but because it coincides with the power spectrum of Brownian motion. Extending the analogy, a rising line would contain all the frequencies but as the large ones dominate, the resulting color would correspond to the various tones of bluish.

Since the seminal work of George Kingsley (Zipf, 1949), the case when α is negative has been of special interest. Zipf examined several corpus in English and found that the frequency of words plotted against their rank constitutes a power law with exponent α = −1. Figure 2 shows the log-log plot of power spectrums following a power law for different values of α.

If α ≈ 0, this means that all the frequencies are present with the same amplitude. If an analogy with the frequencies of electromagnetic radiation is established, then it is possible to state that the color of the time series is white, and the time series is often described as *white noise*. Following the analogy, the case of α = −1 emerges from the presence of all the frequencies but more strongly dominated by low frequencies corresponding to the red region of the color spectrum, and as red plus white gives pink the result is called *pink noise*. Pink noise seems to be ubiquitous in nature; for a number of examples, refer to Bak et al. (1987) and Bak (1996). If the power spectrum decays as *f*^−2^, the low frequencies are even more dominant; for historical reasons, this distribution is called *brown noise* as it formally coincides with Brownian noise (Bak et al., 1987).

## 4. Detrended fluctuation analysis

DFA was introduced by Peng et al. (1995) to analyze non-stationary heartbeat time series. The purpose of the technique is to detect self-similar patterns even if they are embedded in a seemingly non-stationary frame. Furthermore it has the added feature of avoiding the spurious detection of artificial self-similarity due to trending of the probability distribution function. DFA starts with a discrete time series *x*(*i*), for *i* = 1, 2, …, *N* and then this is substituted by the integrated values *y*(*i*) so that a self-similar process is obtained. This integrated time series is defined by

(2) $$\begin{array}{r} y\left(k\right)=\overset{k}{\underset{i\;=\;1}{\sum }}\left(x\left(i\right)-\bar{x}\right), \end{array}$$

where the average value $\bar{x}$ of the times series *x*(*i*) is given by the formula $\bar{x}=\frac{1}{N}\overset{N}{\underset{i\;=\;1}{\sum }}x\left(i\right)$. The next step in DFA consists in measuring the vertical characteristic scale for the integrated time series. This is achieved by dividing *y*(*i*) into *N* boxes of equal length *n*. For each one of these boxes we perform a linear least squares fitting of the data which is referred to as the *local trend* on that box. The ordinate at the straight line is denoted by *y*_*n*_. We note that, more generally, *y*_*n*_ could be the *y* coordinate of a degree *m* polynomial fitting, and by choosing *m* > 1, we would be removing not only constant or linear trends but also higher order trends; to distinguish this particular approach we refer to the resulting method as DFAm, where the value of *m* ≥ 0 represents the degree of the polynomial fitting. We now come to the *detrend* step of DFA: we subtract from *y*(*i*) the linear local trend *y*_*n*_(*i*) for each *n*. Depending on the box size *n*, the characteristic length-scale function for the fluctuations in the integrated and detrended series is:

(3) $$\begin{array}{r} F\left(n\right)=\sqrt{\frac{1}{N}\overset{N}{\underset{k\;=\;1}{\sum }}{\left(y\left(k\right)-y_{n}\left(k\right)\right)}^{2}}. \end{array}$$

Finally, we plot the values of log *n* against the values of logF(n) and observe a linear relationship which indicates the presence of a power law, in other words

(4) $$\begin{array}{r} F\left(n\right)\sim n^{\alpha }. \end{array}$$

The scaling exponent α is calculated as the slope of the line relating logF(n) and log *n*. If α is greater than 0.5, then there are persistent long-range correlations in *x*(*i*). In case α is equal to 1, then we obtain the so called 1/*f* noise (Li and Holste, 2005) a case which has attracted a lot of interest from both physicists and biologists. If 0 < α < 0.5, then this detects anti-correlations in *x*(*i*), that is, large values are expected to be followed by small values and vice versa (Peng et al., 1995). Hence, DFA can be regarded as a methodology for detecting scaling behavior in observational time series that may be affected by non-stationarities. This applies to our case with psychophysiological electric signals.

## 5. Multichannel detrended fluctuation analysis

While DFA allows the analysis of one time series *x*(*i*) in order to assess the long range correlation of the data involved, for example, in the non-stationary heartbeat time series (Peng et al., 1995), or in the non-linear analysis of anesthesia dynamics (Zhang et al., 2001), it is evident that a similar analysis is needed for time series which are sequences of observations consisting of several simultaneous inputs, so as to be able to assess the long range correlation of multichannel data. For example, in our case, we record information from one night polysomnography with several electrodes, because the information registered by a single electrode alone cannot possibly give a complete picture of how the central nervous system is working.

Here we present a generalization of DFA as introduced by Rodriguez et al. (2011) which precisely enables the analysis of multichannel data. Begin with a vector valued time series $\vec{x}\left(i\right)$, that is, for each *i* = 1, 2, …, *N*, we have an *m*-dimensional vector $\vec{x}\left(i\right)=\left(x_{1}\left(i\right),x_{2}\left(i\right),\ldots ,x_{m}\left(i\right)\right)$. We emphasize that *m* represents the number of inputs of each recording. Then the mDFA will basically implement the DFA methodology in each component of the *m*-dimensional time series while manipulating the vector valued time series with the arithmetic of vectors in ℝ^*m*^. So we initiate the process by considering the integrated values of our vector valued time series as in Equation (2), that is,

(5) $$\begin{array}{r} \vec{y}\left(k\right)=\overset{k}{\underset{i\;=\;1}{\sum }}\left(\vec{x}\left(i\right)-\hat{x}\right), \end{array}$$

where $\hat{x}=\frac{1}{N}\overset{N}{\underset{i\;=\;1}{\sum }}\vec{x}\left(i\right)$ is nothing but the vector where each of its components is the average value of the corresponding components of the given vector valued time series $\vec{x}\left(1\right),\vec{x}\left(2\right),\ldots ,\vec{x}\left(N\right)$. This step results in a component-wise self-similar process. We now measure the vertical characteristic scale for the integrated time series in each component. For this purpose, we divide each component of the integrated time series into boxes of equal length *n*. For each one of these boxes and for the data of each component, a linear least squares fitting (called the local trend of the component on that box) is performed. The vector of values of the *y* coordinates of the straight lines is denoted by $\vec{y_{n}}\left(k\right)$. Just as in the DFA process, we detrend the integrated time series $\vec{y}\left(k\right)$ component by component, so that we subtract $\vec{y_{n}}\left(k\right)$. By modifying Equation (3) in such a way that individual contributions for the detrended fluctuations of every vector component *i* = 1, 2, …, *m* are taken into account, we define

(6) $$\begin{array}{r} F\left(n\right)=\sqrt{\frac{1}{N}\overset{N}{\underset{k\;=\;1}{\sum }}{\|\vec{y}\left(k\right)-\vec{y_{n}}\left(k\right)\|}^{2}}. \end{array}$$

When we plot the values of log *n* against the values of logF(n), a linear relationship is observed which indicates the presence of a power law, that is to say

(7) $$\begin{array}{r} F\left(n\right)\sim n^{\alpha }. \end{array}$$

Hence, the scaling exponent α represents the fluctuations and can be approximated as the slope of the line relating logF(n) and log *n*, as in the classic DFA situation.

## 6. Stationarity

Electrophysiological phenomena are typically regarded as complex signals, i.e., non-linear and non-stationary. We therefore assume the time series *x*(*i*) comes from a non-stationary process *X*(*t*) with *E*(*X*(*t*)) = 0 and *E*(*X*^2^(*t*)) < ∞, which admits an evolutionary spectrum as defined in Priestley (1965). The test introduced by Priestley and Subba Rao (Priestley and Subba Rao, 1969) makes use of the concept of evolutionary spectrum (that is, possibly time dependent) of a non-stationary process, and the basis of the method consists essentially in testing the uniformity of that evolutionary spectrum evaluated at a set of different frequencies and instants in time.

For estimating *h*(*t*, ξ), the evolutionary spectral density function (evolutionary SDF) at frequency ξ and time *t*, the “double window” technique is used (Priestley, 1966). The algorithm of the double window is as follows: two functions *w*_τ_ and *g*, referred to as windows, which satisfy the conditions

- $2\pi \int_{-\infty }^{\infty }|g\left(u\right){|}^{2}du=\int_{-\infty }^{\infty }|\Gamma \left(\xi \right){|}^{2}d\xi =1$
- *w*_τ_(*t*) ≥ 0 for all *t*, τ
- *w*_τ_(*t*) → 0 as |*t*| → ∞, for all τ
- $\int_{-\infty }^{\infty }w_{\tau }\left(t\right)dt=1$ for all τ
- $\int_{-\infty }^{\infty }{\left(w_{\tau }\left(t\right)\right)}^{2}dt<\infty$ for all τ
- There exists a constant *C* such that $\lim_{\tau \to \infty }\tau \int_{-\infty }^{t}{|W_{\tau }\left(\lambda )\right|}^{2}d\lambda =C$

where $\Gamma \left(\xi \right)=\int_{-\infty }^{\infty }g\left(u\right)e^{iu\xi }du$, $W_{\tau }\left(\lambda \right)=\int_{-\infty }^{\infty }w_{\tau }\left(t\right)e^{-i\lambda t}\;dt$. The window *g* is used to build an estimator *U* for evolutionary SDF

(8) $$\begin{array}{r} U\left(t,\xi \right)=\overset{t}{\underset{u\;=\;t-T}{\sum }}g\left(u\right)x\left(t-u\right)e^{-i\xi \left(t-u\right)}. \end{array}$$

The estimator *U* is demonstrated (Priestley, 1966) to be asymptotically unbiased (*E*(*U*(*t*, ξ)) ≈ *h*(*t*, ξ)) but inconsistent (*Var*(*U*(*t*, ξ)) ≈ *h*^2^(*t*, ξ)). Thus the second window *w*_τ_(*t*) is used to build a second estimator, $\hat{h}$, which is both asymptotically unbiased and asymptotically consistent

(9) $$\begin{array}{r} \hat{h}\left(t,\xi \right)=\overset{t}{\underset{u\;=\;t-T}{\sum }}w_{\tau }\left(u\right)|U\left(t-u,\xi \right){|}^{2} \end{array}$$

Furthermore, assuming the bandwidth of |Γ(θ)|^2^ to be small compared with the frequency domain bandwidth of *h*(*t*, ξ), or the bandwidth of *W*_τ_(*u*) to be small compared with the time-domain bandwidth of *h*(*t*, ξ), the following approximations can be obtained

- $\text{E}\left(\hat{h}\left(t,\xi \right)\right)\approx h\left(t,\xi \right)$
- $\text{Var}\left(\hat{h}\left(t,\xi \right)\right)\approx \frac{C}{\tau }h^{2}\left(t,\xi \right)\int_{-\infty }^{\infty }{\left|\Gamma \left(\theta \right)\right|}^{4}\;d\theta$

Let $Y\left(t,\xi \right)=\log\left(\hat{h}\left(t,\xi \right)\right)$, then

- E(*Y*(*t*, ξ)) ≈ log(*h*(*t*, ξ))
- $\text{Var}\left(Y\left(t,\xi \right)\right)\approx \frac{C}{\tau }\int_{-\infty }^{\infty }{\left|\Gamma \left(\theta \right)\right|}^{4}d\theta$

It is important to notice that the variance of *Y* is asymptotically independent of both ξ and *t*. Alternatively, we may write

(10) $$\begin{array}{r} Y\left(t,\xi \right)=\log\left(f\left(t,\xi \right)\right)+\varepsilon \left(t,\xi \right), \end{array}$$

where approximately E(ε(*t*, ξ)) = 0 and $\text{Var}\left(\varepsilon \left(t,\xi \right)\right)=\sigma^{2}\frac{C}{\tau }\int_{-\infty }^{\infty }{\left|\Gamma \left(\theta \right)\right|}^{4}$.

Let us choose a set of times *t*_1_, *t*_2_, …, *t*_*I*_ and a set of frequencies ξ_1_, ξ_2_, …, ξ_*J*_ and write *Y*_*i,j*_ = *Y*(*t*_*i*_, ξ_*j*_), *h*_*i,j*_ = *j*(*t*_*i*_, ξ_*j*_) and ε_*i,j*_ = ε(*t*_*i*_, ξ_*j*_) for *i* = 1, 2, …, *I* and *j* = 1, 2, …*J*. Then we obtain a model

(11) $$\begin{array}{r} Y_{ij}=f_{ij}+\varepsilon_{ij} \end{array}$$

where the {ε_*ij*_} can be regarded as uncorrelated if the points (*t*_*i,j*_, ξ_*i,j*_) are sufficiently wide apart. If in addition the number of points are sufficiently large, then it turns out that the {ε_*i, j*_} follow a normal distribution, that is, $\varepsilon_{i,j}\sim N\left(0,\sigma^{2}\right)$. With this assumption, we may rewrite our model as the usual model of the two factor variance analysis, that is, as

(12) $$\begin{array}{r} H_{0}:Y_{i,j}=\mu +\alpha_{i}+\beta_{j}+\gamma_{i,j}+\varepsilon_{i,j} \end{array}$$

For a stationary process, it is fairly straightforward that $E\left(\hat{h}\left(t,\xi \right)\right)\approx h\left(\xi \right)$ is independent of *t*. Therefore, the degree of stationarity may be tested by means of the model

(13) $$\begin{array}{r} H_{1}:Y_{i,j}=\mu +\beta_{j}+\varepsilon_{i,j}. \end{array}$$

against the model *H*_0_ in (12). We now construct the table of the standard analysis of variance for a two factor design, as shown in Table 3.

**Table 3:** Priestley and Rao test variance analyses.

**Item**	**Sum of squares**	**Degrees of freedom**
Between times	$S_{T}=J\overset{I}{\underset{i\;=\;1}{\sum }}{\left(Y_{i,∙}-Y_{∙,∙}\right)}^{2}$	*I*−1
Between frequencies	$S_{F}=I\overset{J}{\underset{j\;=\;1}{\sum }}{\left(Y_{∙,j}-Y_{∙,∙}\right)}^{2}$	*J*−1
Interaction + Residual	$S_{I+R}=\overset{I}{\underset{i\;=\;1}{\sum }}\overset{J}{\underset{j\;=\;1}{\sum }}{\left(Y_{i,j}-Y_{i,∙}-Y_{∙,j}+Y_{∙,∙}\right)}^{2}$	(*I*−1)(*J*−1)
Total	$S_{0}=\overset{I}{\underset{i\;=\;1}{\sum }}\overset{J}{\underset{j\;=\;1}{\sum }}{\left(Y_{i,j}-Y_{∙,∙}\right)}^{2}$	*IJ*−1
Time averages	$Y_{i,∙}=\frac{1}{J}\overset{J}{\underset{j\;=\;1}{\sum }}Y_{i,j}$	
Frequencies averages	$Y_{∙,j}=\frac{1}{I}\overset{I}{\underset{i\;=\;1}{\sum }}Y_{i,j}$	
Total average	$Y_{∙,∙}=\frac{1}{IJ}\overset{I}{\underset{i\;=\;1}{\sum }}\overset{J}{\underset{j\;=\;1}{\sum }}Y_{i,j}$	

The first step of the test uses the statistic $S_{I+R}\sim \sigma^{2}\chi^{2}\left(\left(I-1\right)\left(J-1\right)\right)$, which follows a chi-squared distribution and will be 0 for γ = 0. When the interaction is not significant, we proceed to test the statistic $S_{T}\sim \sigma^{2}\chi^{2}\left(I-1\right)$ which is 0 for β = 0. Stationarity is proved when both interaction and time effect are not significant. For more information see Priestley (1981).

## 7. Results

### 7.1. Neuropsychological testing

As stated above, age and education had an almost perfect negative correlation [*r*_(11)_ = −0.82, *p* < 0.001]. The MMSE and the Neuropsi scores were significantly correlated according to the Pearson correlation coefficient [*r*_(11)_ = 0.65, *p* < 0.01]. Neuropsi scores and age were not correlated [*r*_(11)_ = −0.28, *p* < 0.33].

### 7.2. Polysomnography

As mentioned above, subjects had few sleep complaints reported on the SAST. But on their sleep questionnaires more complaints appeared in the CTRL group: four members of the CTRL group declared their sleep was not very good and three of them good, while all members of the MCI group stated their sleep was good. According to the sleep questionnaire, mean subjective general latency to sleep onset was 21.5 for the CTRL group (range from 10 to 60 min) and 20.7 min (range from 10 to 60 min) for the MCI group, with no significant differences between them [*t*_(11)_ = 0.09, *p* < 0.93]. Nevertheless, according to the Likert scale, the night at the laboratory was evaluated as good (85 and 94%, respectively for the CTRL and MCI group) without significant differences between groups [*t*_(11)_ = 1.57, *p* < 0.14].

The Neurophysiologist found among MCI subjects several cases of suspected RLS and/or fragmented sleep. The intensity of leg movement indexes for the MCI group per hour was abnormally high (range from 16.88 to 70.87 movements per hour). Only two CTRL subjects could be registered for leg movements: one subject had 5.32 sequences of Periodic Leg Movements (PLM) and the other had an index of 46.05 PLM per hour of sleep. The number of awakenings per hour was not abnormal. For the CTRL group, awakenings per hour ranged from 1.8 to 4.5 for all subjects; and the MCI group had from 1.9 to 5.1 awakenings per hour. Nevertheless, when arousals were considered, four CTRL members had a range of arousals per hour of 3.5 to 6.5 and three of them from 13.23 to 24.04. Instead, two subjects from the MCI group had a range of 2.6 to 7.6 arousals per hour, while four of them had 12.59 to 35.9 arousals per hour. The number of arousals reached statistical significance, the frequency of arousals for the MCI group being higher than for the CTRL group (chi = 6.26, df = 1, *p* < 0.01). In addition, another subject belonging to the MCI group had a diminished latency to REM sleep suggestive of narcolepsy. A subject of the CTRL group presented a diminished latency to REM sleep suggestive of depression but the clinical assessment ruled out that possibility. Despite those results, there were no significant differences regarding TST, percentages of sleep stages, efficiency, latencies to sleep onset or to REM sleep for the groups. Table 4 shows these polysomnographic results, the epochs recorded as “wakefulness,” the index of wakefulness per hour of sleep, and the overall number of arousals and arousals per hour.

**Table 4:** Polysomnographic sleep measures for each group.

	**CTRL**		**MCI**			
	**Mean**	***SD***	**Mean**	***SD***	***t***	***p***
TST (min)	372.86	72.04	347.91	89.83	0.55	0.58
N1/TST	6.91	4.87	9.33	5.80	0.81	0.43
N2/TST	48.41	10.48	42.70	20.77	0.64	0.53
N3/TST	17.96	8.70	19.13	9.44	0.23	0.82
REM/TST	12.86	4.28	11.92	3.51	0.42	0.67
WASO	11.65	3.65	9.83	11.10	0.41	0.68
Sleep efficiency (%)	83.24	4.71	85.27	11.40	0.43	0.67
Latency to sleep (min)	12.98	9.06	10.18	11.16	0.49	0.62
Latency to REM (min)	101.85	39.50	78.57	41.85	1.03	0.32
**Wakefulness and arousals -n**					**χ**^**2**^	***p***
Wakefulness	156		147		0.26	0.60
Wakefulness per hr	22.3		19.70		0.16	0.68
Arousals	588		677		6.26	**0.01**
Arousals per hr	77.95		88.11		0.62	0.43

*TST, Total Sleep Time; N1, Stage 1; N2, Stage 2; N3, Stage 3+4; REM, Rapid Eye Movement sleep; WASO, Wakefulness After Sleep Onset; MCI, Mild Cognitive Impairment; CTRL, Control. Student t-tests, p < 0.05. Significant results for ANOVA tests are indicated in bold*.

### 7.3. DFA and mDFA

The percentages of NREM for N1, N2 and N3 stages for the CTRL group were 10, 51.4, and 38.5%, respectively; likewise, for the MCI group, 1.6, 70, and 28.3%, respectively. None of the frequencies of the ten NREM epochs subjected to the quantitative analyses for the DFA and mDFA showed significant differences between groups (chi = 1.56, df = 1, *p* < 0.21 for N1; chi = 0.46, df = 1, *p* < 0.49 for N2; chi = 2.27, df = 1, *p* < 0.13 for N3).

**Figure 3:**
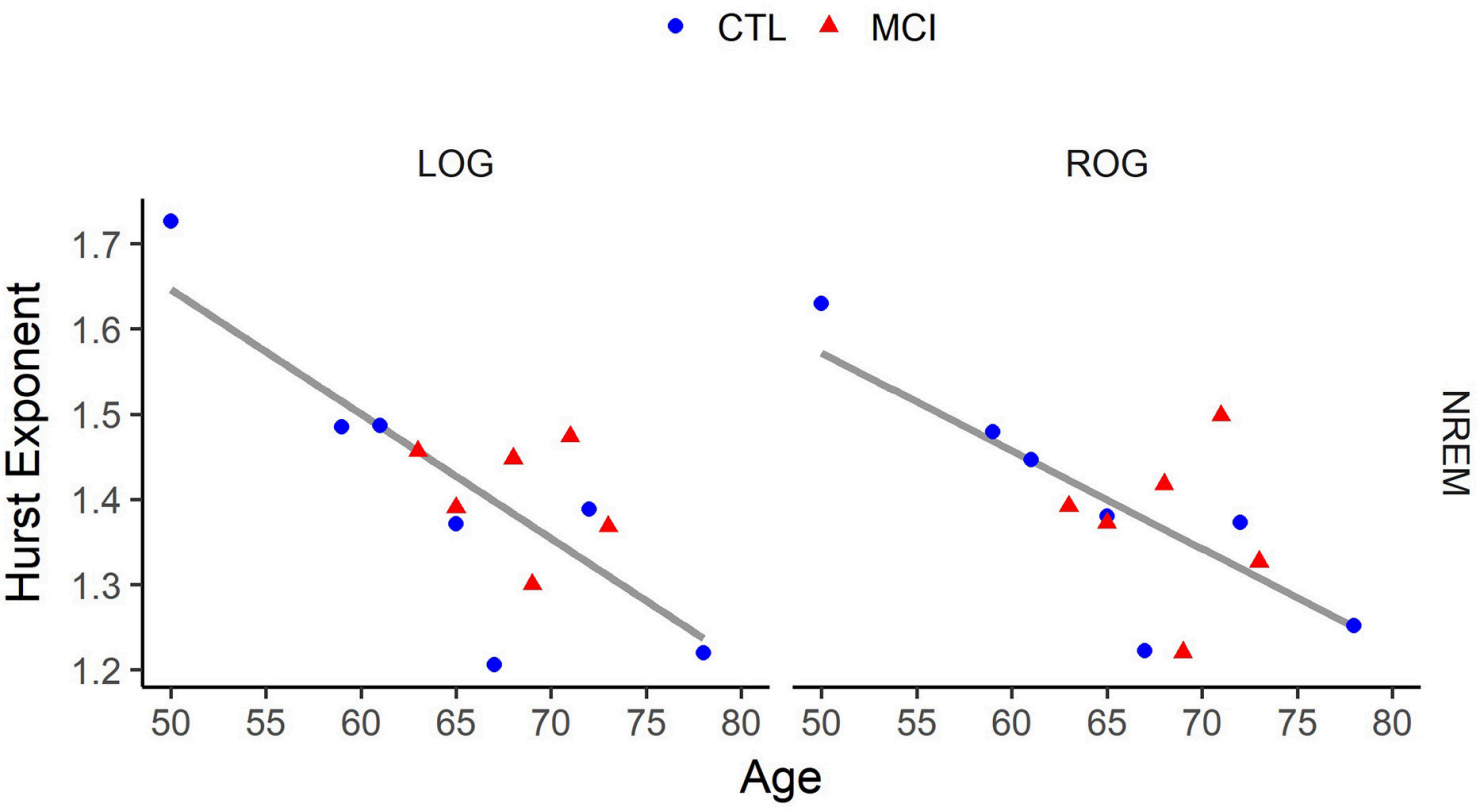
Correlation of age vs. Hurst values in Non-Rapid Eye Movement (NREM) Sleep for each eye signal. LOG, Left Oculogram; ROG, Right Oculogram; CTRL, Control group; MCI, Mild Cognitive Impairment group. Significant results for ANOVA tests are indicated in bold.

Hurst mean values in NREM and age were negatively correlated at LOG and ROG derivations [*r*_(11)_ = −0.78, *p* < 0.001; *r*_(11)_ = −0.70, *p* < 0.001], for LOG and ROG, respectively], as can be seen in Figure 3.

On the other hand, education was positively correlated with Hurst mean values in NREM sleep at the same derivations, LOG and ROG [*r*_(11)_ = 0.68, *p* < 0.01; *r*_(11)_ = 0.61, *p* < 0.02, respectively]. In addition, a significant positive correlation between the Neuropsi scores and the Hurst mean exponents of NREM sleep was found at the left posterior network [*r*_(11)_ = 0.55, *p* < 0.04].

For REM sleep, Hurst mean values and education were negatively correlated at the EMG [*r*_(11)_ = -0.67, *p* < 0.02].

**Figure 4:**
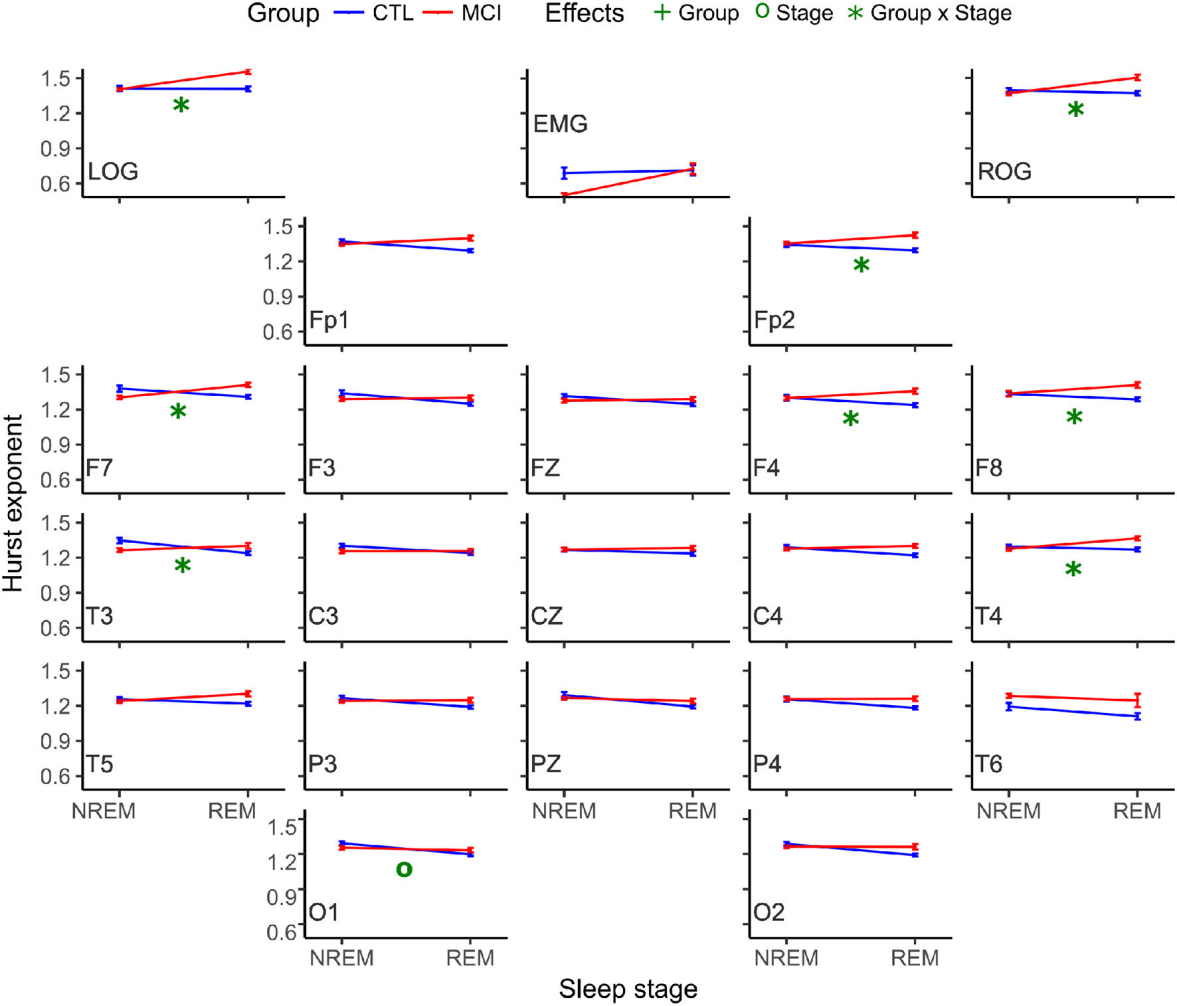
Mean Hurst values for each group and stage. NREM, Non-REM Sleep; REM, Rapid Eye Movement sleep; LOG, Left Oculogram; ROG, Right Oculogram; EMG, Electromyogram; CTRL, Control group; MCI, Mild Cognitive Impairment group.

ANOVA results considering only the mean of the Hurst values for the ten NREM and the mean of the ten REM epochs were not significant. Nevertheless, the comparison considering the ten Hurst values of the NREM and REM epochs rendered one difference in stages at O1. Both Hurst values decreased from NREM to REM. Interactions in the ANOVA tests for the monopolar channels were found at LOG and ROG channels and frontotemporal derivations (FP2, F8, F7, F4, T4, and T3) and in all cases, the means were higher in REM sleep stages for the MCI group (Table 5 and Figure 4).

**Table 5:** Scaling results for each group in the transition from NREM to REM at individual derivations.

	**CTRL**		**MCI**		**Mixed ANOVA**					
	**NREM**	**REM**	**NREM**	**REM**	**Group**		**Stage**		**Group × stage**	
					**Df** = **1, 22**		**Df** = **1, 22**		**Df** = **1, 22**	
	**Mean *(SD)***	**Mean *(SD)***	**Mean *(SD)***	**Mean *(SD)***	***F***	***p***	***F***	***p***	***F***	***p***
FP2	1.34 (0.15)	1.29 (0.13)	1.35 (0.11)	1.42 (0.17)	1.31	0.276	0.10	0.754	5.81	**0.035**
FP1	1.37 (0.15)	1.29 (0.12)	1.35 (0.12)	1.40 (0.16)	0.56	0.471	0.37	0.555	4.55	0.056
F8	1.33 (0.16)	1.29 (0.14)	1.34 (0.19)	1.41 (0.18)	0.71	0.417	0.11	0.750	6.35	**0.029**
F7	1.38 (0.21)	1.31 (0.13)	1.30 (0.11)	1.41 (0.14)	0.04	0.852	0.12	0.734	7.49	**0.019**
F4	1.30 (0.19)	1.24 (0.15)	1.30 (0.12)	1.36 (0.17)	0.54	0.476	0.11	0.742	8.79	**0.013**
F3	1.34 (0.21)	1.25 (0.14)	1.29 (0.12)	1.30 (0.15)	0.00	0.996	3.11	0.106	4.23	0.064
T4	1.30 (0.12)	1.27 (0.14)	1.28 (0.12)	1.37 (0.13)	0.38	0.549	1.54	0.241	6.82	**0.024**
T3	1.35 (0.20)	1.24 (0.13)	1.26 (0.13)	1.30 (0.19)	0.02	0.890	1.75	0.213	5.30	**0.042**
C4	1.29 (0.17)	1.22 (0.13)	1.28 (0.10)	1.30 (0.13)	0.35	0.568	1.22	0.293	3.93	0.073
C3	1.30 (0.15)	1.24 (0.14)	1.26 (0.13)	1.26 (0.14)	0.06	0.809	2.03	0.182	1.82	0.205
T6	1.19 (0.26)	1.11 (0.22)	1.29 (0.14)	1.25 (0.44)	0.86	0.374	2.15	0.171	0.25	0.626
T5	1.26 (0.13)	1.22 (0.14)	1.24 (0.12)	1.30 (0.16)	0.28	0.609	0.07	0.803	3.27	0.098
P4	1.26 (0.17)	1.18 (0.11)	1.26 (0.12)	1.26 (0.16)	0.37	0.557	2.97	0.113	2.88	0.118
P3	1.27 (0.17)	1.19 (0.11)	1.24 (0.11)	1.25 (0.16)	0.06	0.805	2.61	0.135	2.98	0.112
O2	1.29 (0.13)	1.19 (0.10)	1.26 (0.10)	1.26 (0.17)	0.20	0.660	3.41	0.092	2.70	0.129
O1	1.29 (0.13)	1.20 (0.12)	1.25 (0.12)	1.23 (0.16)	0.00	0.980	5.66	**0.037**	1.94	0.191
FZ	1.32 (0.15)	1.25 (0.14)	1.28 (0.12)	1.29 (0.15)	0.00	0.986	2.32	0.156	4.13	0.067
CZ	1.27 (0.13)	1.24 (0.16)	1.27 (0.12)	1.28 (0.14)	0.14	0.717	0.30	0.594	1.23	0.291
PZ	1.29 (0.21)	1.19 (0.12)	1.27 (0.11)	1.24 (0.16)	0.03	0.872	3.67	0.082	1.07	0.322
LOG	1.41 (0.18)	1.41 (0.17)	1.41 (0.09)	1.56 (0.18)	1.07	0.324	3.93	0.073	4.96	**0.048**
ROG	1.40 (0.16)	1.37 (0.17)	1.37 (0.12)	1.51 (0.18)	0.76	0.401	2.20	0.166	6.19	**0.030**
EMG	0.69 (0.38)	0.71 (0.35)	0.50 (0.12)	0.73 (0.33)	0.24	0.633	1.79	0.213	1.33	0.278

*CTRL, Control group; MCI, Mild Cognitive Impairment group; REM, Rapid Eye Movement sleep; NREM, Non-REM sleep; LOG, Left Oculogram; ROG, Right Oculogram; EMG, Electromyogram. Significant results for ANOVA tests are indicated in bold. In each case, the Greenhouse-Geisser's correction for repeated measures was employed*.

**Figure 5:**
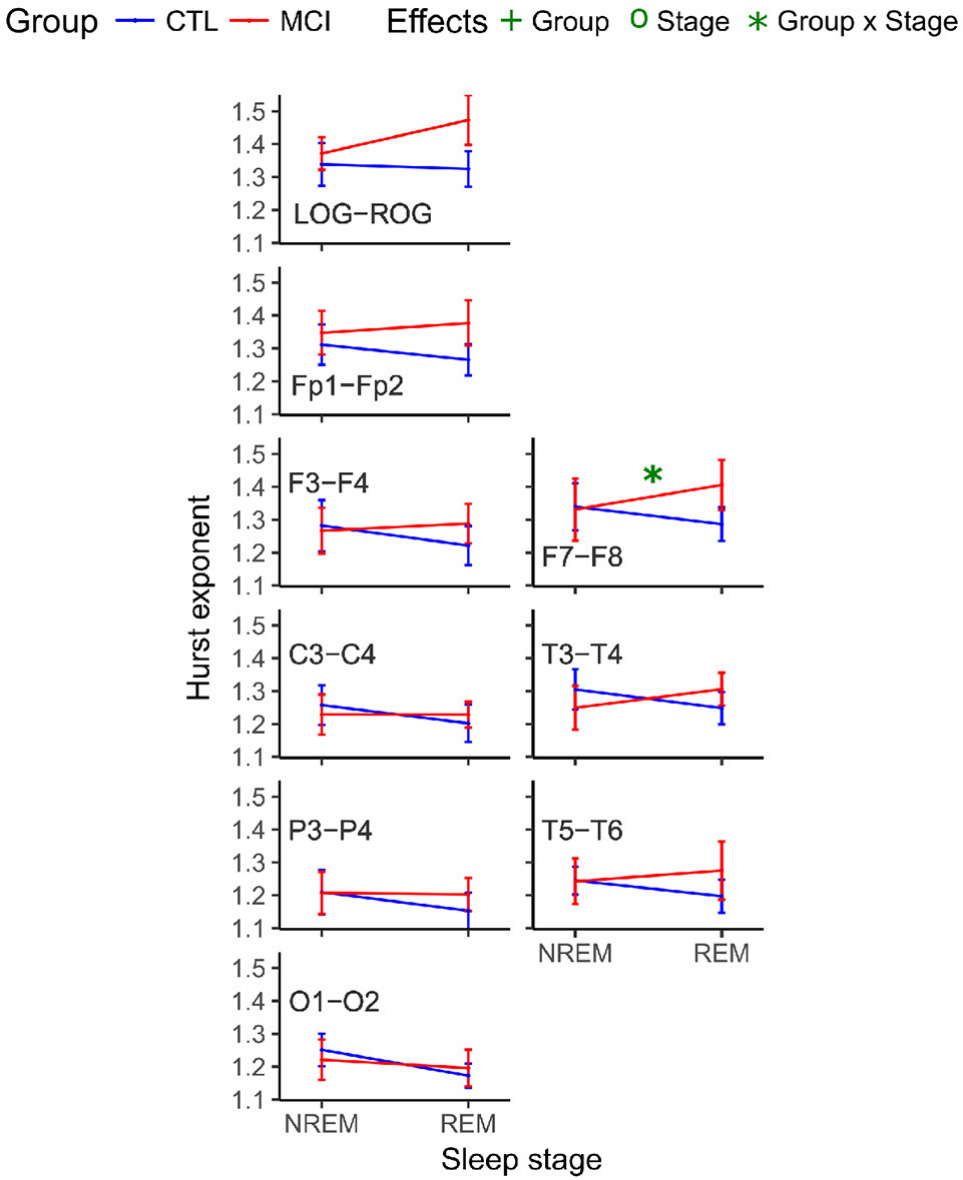
Mean Hurst values for each group and stage for interhemispheric networks. NREM, Non-Rapid Eye Movement sleep; REM, Rapid Eye Movement sleep; LOG, Left Oculogram; ROG, Right Oculogram; CTRL, Control group; MCI, Mild Cognitive Impairment group.

Another interaction at the interhemispheric relationship was significant at the F7-F8 pair of derivations, following the same tendency of being higher for the MCI group (Table 6 and Figure 5).

**Table 6:** Scaling results for each group in the transition from NREM to REM at anteroposterior and posterior networks.

	**CTRL**		**MCI**		**Mixed ANOVA**					
	**NREM**	**REM**	**NREM**	**REM**	**Group**		**Stage**		**Group × stage**	
					**Df** = **1, 22**		**Df** = **1, 22**		**Df** = **1, 22**	
	**Mean *(SD)***	**Mean *(SD)***	**Mean *(SD)***	**Mean *(SD)***	***F***	***p***	***F***	***p***	***F***	***p***
FP1-FP2	1.31 (0.17)	1.27 (0.14)	1.35 (0.16)	1.38 (0.17)	0.87	0.371	0.11	0.747	1.24	0.290
F7-F8	1.34 (0.20)	1.29 (0.15)	1.33 (0.23)	1.41 (0.19)	0.31	0.590	0.05	0.834	5.03	**0.047**
F3-F4	1.28 (0.21)	1.22 (0.16)	1.27 (0.17)	1.29 (0.15)	0.08	0.786	0.86	0.373	2.87	0.118
T3-T4	1.30 (0.18)	1.25 (0.14)	1.25 (0.16)	1.31 (0.13)	0.00	0.991	0.03	0.872	4.57	0.056
C3-C4	1.26 (0.16)	1.20 (0.16)	1.23 (0.14)	1.23 (0.11)	0.00	0.990	2.08	0.177	1.73	0.216
T5-T6	1.25 (0.13)	1.20 (0.15)	1.24 (0.17)	1.28 (0.21)	0.20	0.660	0.13	0.722	1.83	0.204
P3-P4	1.21 (0.18)	1.15 (0.15)	1.21 (0.15)	1.20 (0.13)	0.08	0.776	1.86	0.199	1.16	0.305
O1-O2	1.25 (0.13)	1.17 (0.13)	1.22 (0.15)	1.20 (0.14)	0.00	0.958	3.64	0.083	0.88	0.368
LOG-ROG	1.34 (0.18)	1.32 (0.16)	1.37 (0.14)	1.47 (0.18)	1.36	0.268	0.98	0.343	2.10	0.176
FP2-P4	1.27 (0.18)	1.22 (0.15)	1.26 (0.15)	1.33 (0.17)	0.32	0.583	0.17	0.685	7.22	**0.021**
FP1-P3	1.28 (0.17)	1.23 (0.12)	1.26 (0.15)	1.31 (0.14)	0.14	0.712	0.02	0.877	4.39	0.060
O2-P4-T4	1.25 (0.14)	1.22 (0.15)	1.25 (0.16)	1.27 (0.14)	0.09	0.771	0.09	0.775	0.87	0.370
O1-P3-T3	1.28 (0.16)	1.21 (0.15)	1.20 (0.15)	1.21 (0.13)	0.37	0.557	1.40	0.262	2.43	0.147

*CTRL, Control group; MCI, Mild Cognitive Impairment group; REM, Rapid Eye Movement sleep; NREM, Non-REM sleep; LOG, Left Oculogram; ROG, Right Oculogram; EMG, Electromyogram. Significant results for ANOVA tests are indicated in bold. In each case, the Greenhouse-Geisser's correction for repeated measures was employed*.

**Figure 6:**
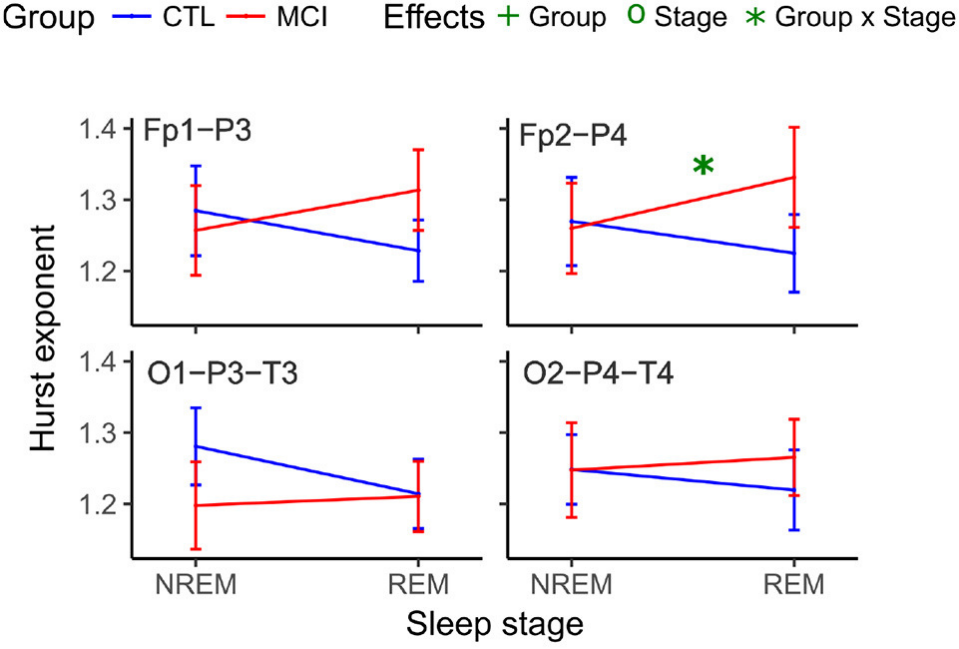
Mean Hurst values for each group and stage at anteroposterior and posterior networks. NREM, Non-Rapid Eye Movement sleep; REM, Rapid Eye Movement sleep; LOG, Left Oculogram; ROG, Right Oculogram; CTRL, Control group; MCI, Mild Cognitive Impairment group.

Likewise, the scaling values for the right frontoparietal network showed an interaction and increased for the MCI group and decreased for the CTRL group (Table 6 and Figure 6).

### 7.4. Stationarity

**Figure 7:**
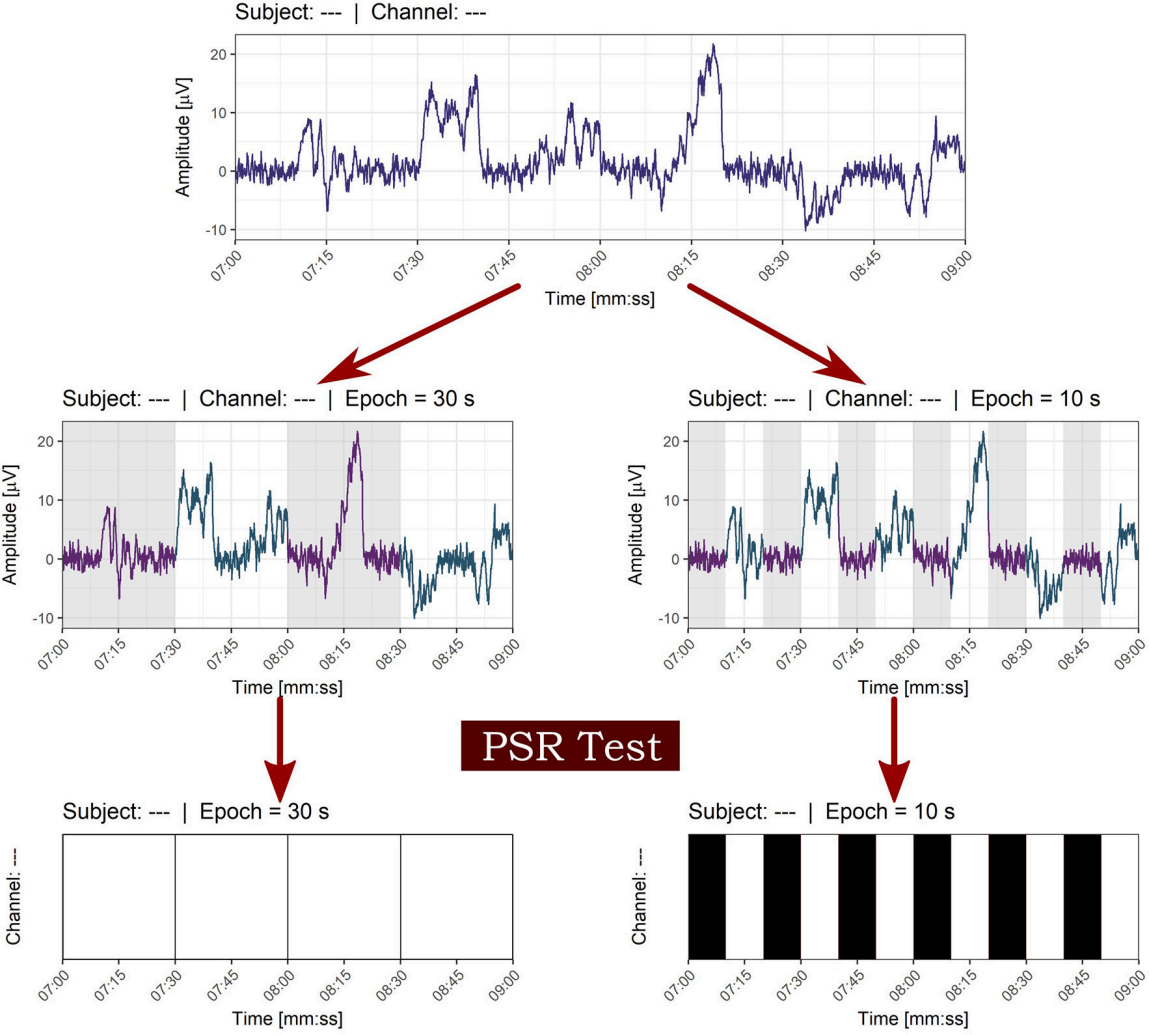
Effect of window size on the classification of stationarity. The epoch of 30 s in contrast with the 10 seconds window was chosen in order to avoid local stationarity of small size windows. White bars indicate non-stationarity and black bars stationarity in time series of epochs.

Each of the NREM and REM epochs was classified as stationary or non-stationary by using the described methodology stated above and considering a 30- seconds epoch was a better estimate of the degree of stationarity than a 10-seconds epoch, as can be seen in Figure 7.

**Figure 8:**
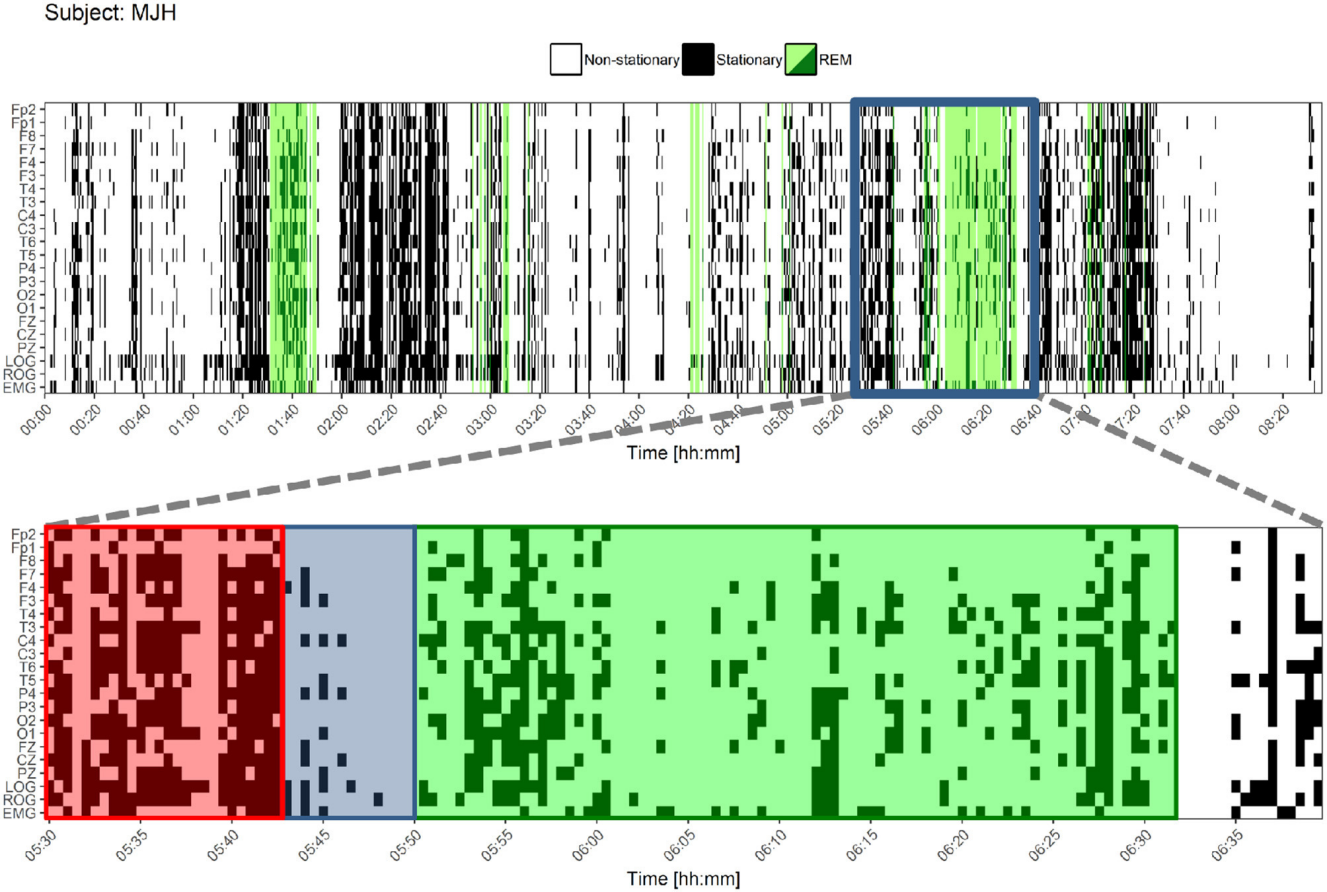
Stationary patterns according to the Priestley and Subba Rao Tests during the whole night for one control subject. At the upper figure, each black or white point represents an epoch. Black epochs indicate a greater degree of stationarity and white ones a lesser degree of stationarity. REM sleep is colored in green. Below, a closer look is shown. A pattern is found before REM sleep onset and during the ongoing REM sleep: a higher degree of stationarity (red) that becomes suddenly less stationary (blue) and adopts another non-stationary pattern for REM sleep (green). LOG, Left Oculogram; ROG, Right Oculogram; EMG, Electromyogram.

Both types of classified epochs were represented visually for the whole night register in Figure 8.

**Figure 9:**
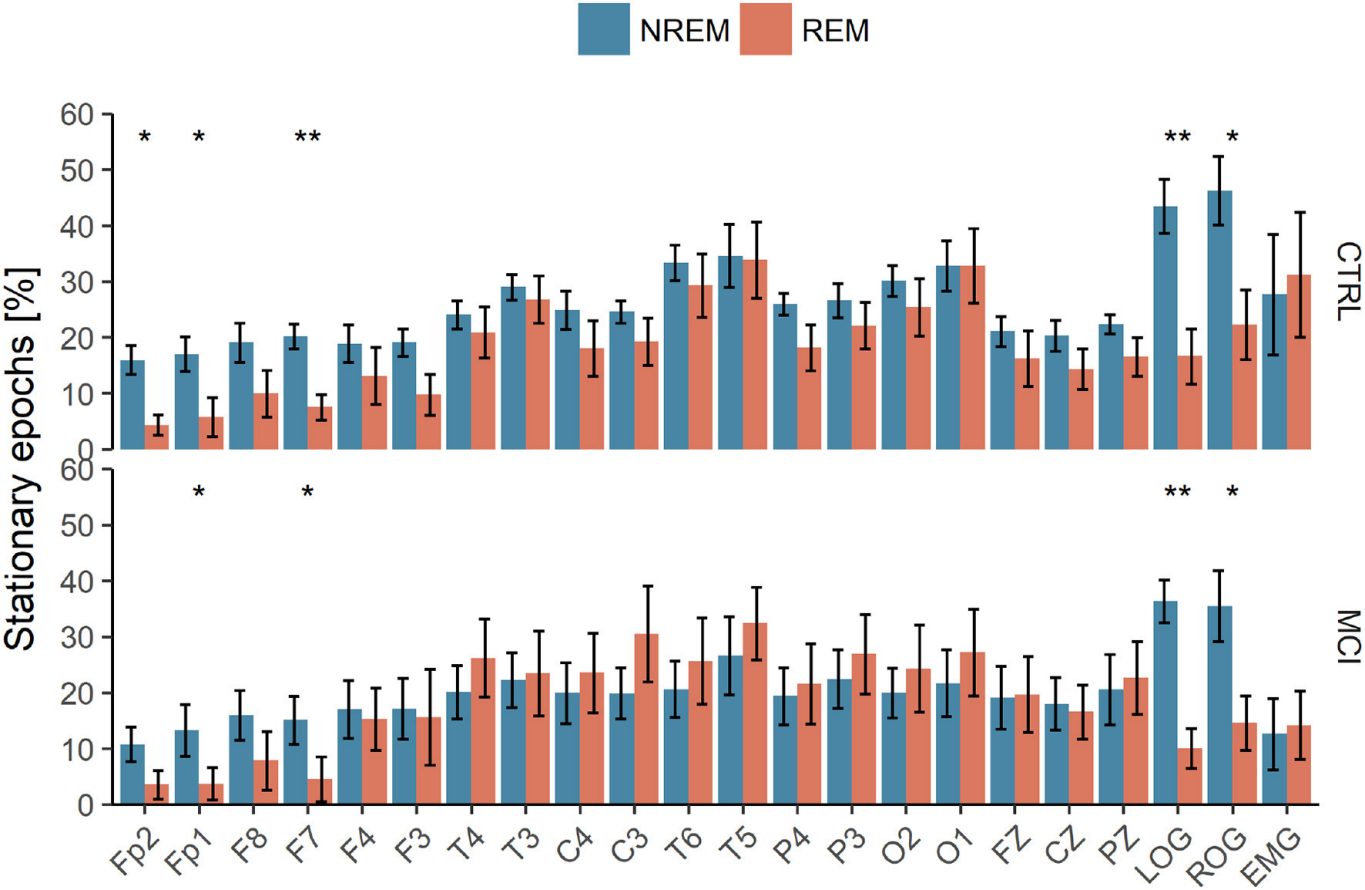
Polysomnographic recordings of the whole night subjected to Priestley and Subba Rao Tests for stationarity analyses. NREM, Non-Rapid Eye Movement Sleep; REM, Rapid Eye Movement Sleep; LOG, Left Oculogram; ROG, Right Oculogram; CTRL, Control Group; MCI, Mild Cognitive Impairment Group. One or two asterisks mean *p* < 0.05 and *p* < 0.001, respectively, according to Wilcoxon tests.

The quantity of those stationary epochs did not show significant correlations with age, neither with MMSE nor Neuropsi scores. Also, no significant differences between groups nor sleep stages were found considering the ten values from each stage; this is believed to be due to a small-sample effect. The methodology was repeated for the whole night PSG register and similar analyses were performed. There was a great variability, as shown in the Table S1 of the Supplementary Material. Nevertheless, Wilcoxon tests were significant for each group in frontal (FP2, FP1, and F7) and LOG and ROG channels. For the CTRL group, F3 was also significant, as can be seen in Figure 9.

These results suggest that (1) the differences between REM and NREM can be effectively traced using simple techniques such as weak stationarity detection (Figure 8), and (2) there are real differences between groups, though not enough to be detected statistically by this method.

## 8. Discussion

REM sleep enhances memory and attention processes by cholinergic inputs (Braun et al., 1997) via pontine (Datta et al., 2004) and basal forebrain structures (Blake and Boccia, 2017). During normal aging and especially during pathological aging, attention and memory processes become more vulnerable, and cholinergic neurons are mostly affected (Schliebs and Arendt, 2011). Aging affects various anatomic structures resulting in a loss of dendritic arbor in cortical neurons that show degradation in their structural fractal complexity (Lipsitz and Goldberger, 1992).

Non-linear dynamics and complex systems appear to be well suited to explain these phenomena (Babloyantz and Destexhe, 1986). There is a number of reports specifically addressing EEG signals during sleep: from the theoretical quantification of the effects of non-linearity (Aeschbach and Borbérly, 1993; Fell et al., 1993) to characterization of some pathologies (Röschke et al., 1995). Recent advances in nonlinear dynamics have pointed toward the relevance of using the framework of power-laws and associated tools to extract information from the EEGs (Lee et al., 2004), nevertheless, subjects under investigation have been few; only healthy subjects or subjects with sleep disorders as apnea, insomnia or narcolepsy have been studied, and non-linear methods have been heterogeneous.

Recent work showed a generalization of the DFA, named multivariate DFA (MVDFA) (Xiong and Shang, 2017). This analysis is very similar to the one developed by our group and referred to in this paper as mDFA (Rodriguez et al., 2011). Xiong and Shang (2017) showed the validity of the proposed MVDFA illustrated by numerical simulation on synthetic multivariate processes as well as on stock indices in Chinese and U.S. stock markets. Furthermore, these authors calculated the DFA of a single time series and showed that MVDFA is related to the average DFA of each time series. In this research we used mDFA to show interhemispheric, anteroposterior and posterior network behavior between EEG recordings and DFA of a single channel to validate differences between the CTRL and the MCI groups.

Lee et al. (2004) calculated the Hurst exponent of the recordings of normal sleep stages of six healthy subjects against the Hurst exponent of six recordings of apnea from MIT/BIH polysomnography database. The scaling exponents of apnea were found to be lower than those of healthy subjects.

Acharya et al. (2005) computed several parameters, including the Hurst exponent but not through DFA. They worked out their analysis with eight EEG data from the sleep-EDF database from the PhysioBank, a data resource.

Weiss et al. (2009) based their study on the data from ten subjects, a similar number of subjects to those studied in the present research. Weiss et al. (2011) confirmed their previous results concerning Hurst values but now with twenty two participants. They added correlation analyses between Hurst exponents and the range of fractal spectra to strengthen their previous results through the use of fractal analysis to emphasize known phenomena of human sleep, in this case by proving that fractal range was a better estimating measure for classifying sleep stages.

Kumar et al. (2012) have proposed a pharmacological and neurophysiological model to reveal how the transition from wakefulness to NREM and REM sleep occurs. The transition has been explored in younger adults or in people with sleep disorders by means of mathematical analyses of one of the markers of REM sleep. In Kishi et al. (2011), a control night was compared with a night after a dose of risperidone, but only latencies and percentages of EEG stages were obtained. In another work in healthy young subjects an abrupt change in spectral analysis for two broad bands was observed on the EMG: one from 24 to 28 Hz and the other from 28 to 32 Hz (Rosales-Lagarde et al., 2009). Also, Bliwise et al. (1974) searched for EMG changes in 5 young female adults and discovered that the lowest tonic levels in EMG occurred just before REM sleep, increasing for subsequent periods of NREM sleep and decreasing again before the subsequent REM period. Hadjiyannakis et al. (1997) followed the three REM sleep markers, did spectral analysis of the EEG in a larger window than the above mentioned study of Rosales-Lagarde et al. (2009) and concluded that neither in normal controls nor in narcoleptics, abrupt modifications in the power density of the frequencies were observed.

Nevertheless, the NREM to REM transition has not been explored by non-linear analysis with the exception of Bizzotto et al. (2010) who used Markov-chain models in insomnia patients, but to our knowledge no paper has presented a non-linear analysis in the NREM to REM transition in MCI subjects.

In our research, the three indicators of sleep were tested. There were selective differences in the transition from NREM to REM sleep for each group.

On the EEG, while the CTRL group, with preserved memory, attention and executive functions diminished its signal structure in frontotemporal areas from NREM to REM sleep, the MCI group had higher values suggestive of a tendency toward Brownian noise, strongly dominated by low frequencies, a result that had to be confirmed later using spectral analysis. In this matter, CTRL results agree with the findings of Weiss et al. (2009), because lower Hurst values were found in frontal regions in REM sleep. This is in accordance to several studies that refer to an anteroposterior gradient using metabolic techniques in resting states, indicating a greater hypofrontality relative to age (Moeller et al., 1996), but also during REM sleep. Only the left occipital area distinguished between stages. Likewise, Weiss et al. (2009) found differences for the Hurst values from NREM4 to REM in that site. The mDFA rendered a frontopolar relationship, because while the CTRL group diminished its scaling exponents, the MCI group increased them.

Inter-individual connectivity has been proved to distinguish heteromodal cortices from primary areas (Mueller et al., 2013) and in this work has been associated with group differences in cognitive functioning in the NREM and REM transition.

A dissimilar pattern among interhemispheric coupling, anteroposterior and posterior areas (Pérez-Garci et al., 2001; Corsi-Cabrera et al., 2003) was not found. Instead, the individual scaling exponents and the networks followed a greater Hurst value for the MCI group.

From NREM to REM, at both right and left EOG, the MCI group increased its signal structure toward Brownian noise. The correlations with Hurst values of the EOGs in NREM sleep rendered a negative association with age and a positive one with education. Also, in NREM sleep, the left posterior network was positively correlated with Neuropsi scores, meaning this network is sensitive for measuring cognitive impairment.

Muscle activity at REM sleep was negatively correlated with education. Van der Hiele et al. (2011) found more theta activity, less alpha reactivity, and more frontal EMG in Alzheimer's patients than in controls. Increased EMG activity indicated more cognitive impairment and more depressive complaints. Also, Chen et al. (2011) used root mean square and frequency peak analysis and found greater values for MCI patients. In the present study, contrary to EEG and EOG results, EMG values tended to have scaling exponents of white noise, or, as stated above, anti-correlations: large values are expected to be followed by small values and vice versa.

Regarding the great number of participants with RLS, the latter could not be diagnosed, because all MCI subjects stated their sleep was good and were practically without sleep complaints. Periodic RLS episodes are followed by increases in power, heart beat and arousals (Sieminski et al., 2017). Ferri et al. (2015) concluded that RLS is connected, but not in a simple causal relation, to arousals. The conclusion of Frauscher et al. (2014) refers to a high rate of motor events even in normal subjects. These authors found PLM especially in N1 stage, being the median of 5 per hour. The great number of arousals can help to explain group differences, because subjective complaints, both of memory and sleep, were minimal. Changes of beta-amyloid ocurring in sleep disorders point toward the disruption of NREM sleep and, in particular, of SWS (Brown et al., 2016; Cellini, 2017). Potential underestimation of memory and sleep may affect these MCI subjects more than CTRLs. Gender effects could have influenced these results (Nikulin and Brismar, 2005), because almost all the CTRL group were women and most of the MCI subjects were men. In future works this factor must be controlled for.

The arbitrary exception for the degree of detection of stationarity when using the whole-night register, instead of a few NREM and REM epochs, was motivated mainly to counter the small-sample effect, but it was also preferred for being one of the fastest algorithms to compute (Nason, 2013). Previous results showed that this technique can be used for detecting sleep stages for OAs (Rosales-Lagarde et al., 2017), so these new results indicate that such detection is still valid for some cognitive impaired OAs.

Finally, it must be highlighted that larger samples are needed to confirm the present findings.

## 9. Conclusion

At the NREM to REM transition, executive functioning in MCI subjects was associated with brown noise in frontotemporal and LOG and ROG scaling exponents. On the EEG, both for DFA and mDFA, MCI OAs performing poorly on memory, attention and executive functions increased their Hurst values toward Brown noise from NREM to REM stages, while the CTRL group followed an opposite direction. On the EOG, both groups increased their Hurst values, and again the MCI group came nearer to its fractal breakdown. Muscle scaling values were lower than cerebral and eye movement Hurst values. Stationary differences were found at the whole register for the distinction of stages within the groups. Given the small size of the samples, any conclusion should be considered as preliminary and to confirm this data larger studies are needed.

## Ethics statement

This study was carried out in accordance with the recommendations of La Coordinación de Investigación of the Instituto de Ciencias de la Salud at the Universidad Autónoma del Estado de Hidalgo. The protocol was approved by El Comité de Ética de Investigación. All subjects gave written informed consent in accordance with the Declaration of Helsinki.

## Author contributions

AR-L, GV-T, and CM-A contributed the neuropsychological testing and selection of the OAs. AR-L and GV-T conducted the PSGs. AR-L and LC-R scored the PSGs. AR-L wrote most of the paper related to the clinical, neuropsychological and psychophysiological sections. ER-T organized VG-M and JE-A to do the Hurst and stationarity analysis, respectively. AR-L, JE-A, and ER-T did the statistical analysis, the tables and figures. ER-T, BI-O and PM wrote the mathematical section approaches of the analysis and helped with the format and the discussion. JL-N and JP-S provided administrative and technical support to carry out the sleep measurements at the UAEH and helped with the references.

### Conflict of interest statement

The authors declare that the research was conducted in the absence of any commercial or financial relationships that could be construed as a potential conflict of interest.
