# Supplementary material for: The Color of Noise and Weak Stationarity at the NREM to REM Sleep Transition in Mild Cognitive Impaired Subjects
Source: Front Psychol. 2018 Jul 17;9:1205. doi: 10.3389/fpsyg.2018.01205 (PMC6056768; doi:10.3389/fpsyg.2018.01205)
Supplement: Supplementary file 1 [file Table_1.pdf]

The Color of Noise and Weak Stationarity at the NREM to REM sleep transition in Mild Cognitive Impaired subjects

Alejandra Rosales-Lagarde*, Erika Elizabeth Rodriguez-Torres, Benjamín A. Itzá-Ortiz, Pedro Miramontes, Génesis Vázquez-Tagle, Julio Cesar Enciso-Alva, Valeria García-Muñoz, Lourdes Cubero-Rego, José Esael Pineda-Sánchez, Claudia Isabel Martínez-Alcalá, Jose Socrates Lopez-Noguerola.

1 SUPPLEMENTARY DATA

Details of the custom code for the MATLAB to calculate multichannel Detrended Fluctuation Analyses on EEG recordings. This code can be modified to calculate DFA of one single EEG recording.

```
 ********* Begin  DFAmain2 ***********

 function [Fn]=DFAmain2(DATA1,DATA2)

* DATA1 and DATA2 are EEG time series
* n box sizes up to 30 seconds in points
* Fn fluctuations
n=[4,5,6,7,8,9,10,11,12,13,15,16,17,19,21,23,25,27,29,32,35,...
   1448,1579,1722,1878,2048,2233,2435];

N1=length(n);
Fn=zeros(N1,1);
 for i=1:N1
     Fn(i)=DFA2(DATA1,DATA2,n(i),1); * call a second function DFA2
 end
return

************END DFAmain2 **************


************* BEGIN DFA2 ****************************
 function output1=DFA2(DATA1,DATA2,winlength,order)

 *Fn for the first EEG recording

       N1=length(DATA1);
       n=floor(N1/winlength);
       N11=n*winlength;
```

```
    y1=zeros(N11,1);
    Yn1=zeros(N11,1);

    fitcoef1=zeros(n,order+1);
         mean11=mean(DATA1(1:N11));
         for i=1:N11

             y1(i)=sum(DATA1(1:i)-mean11);
         end
         y1=y1';
         for j=1:n
             fitcoef1(j,:)=polyfit(1:winlength,...
                           y1(((j-1)*winlength+1):j*winlength),...
                           order);
         end

         for j=1:n
             Yn1(((j-1)*winlength+1):j*winlength)=...
                         polyval(fitcoef1(j,:),1:winlength);
         end

%Fn for the second EEG recording

    N2=length(DATA2);
    n=floor(N2/winlength);
    N12=n*winlength;
    y2=zeros(N1,1);
    Yn2=zeros(N1,1);

    fitcoef2=zeros(n,order+1);
         mean12=mean(DATA2(1:N12));
         for i=1:N12

             y2(i)=sum(DATA2(1:i)-mean12);
         end
         y2=y2';
         for j=1:n
             fitcoef2(j,:)=polyfit(1:winlength,...
             y2(((j-1)*winlength+1):j*winlength),...
             order);
         end

         for j=1:n
             Yn2(((j-1)*winlength+1):j*winlength)=...
                         polyval(fitcoef2(j,:),1:winlength);
```

```
          end

          sum1=sum(((y1'-Yn1)).^2);

          sum2=sum(((y2'-Yn2)).^2);

          sum1=sqrt((sum1+ sum2)/N11);
          output1=sum1;


%%%%%%%%%%%END DFA2 %%%%%%%%%%%%%%%%%%%
```

2 SUPPLEMENTARY FIGURES AND TABLES

2.1 Figure

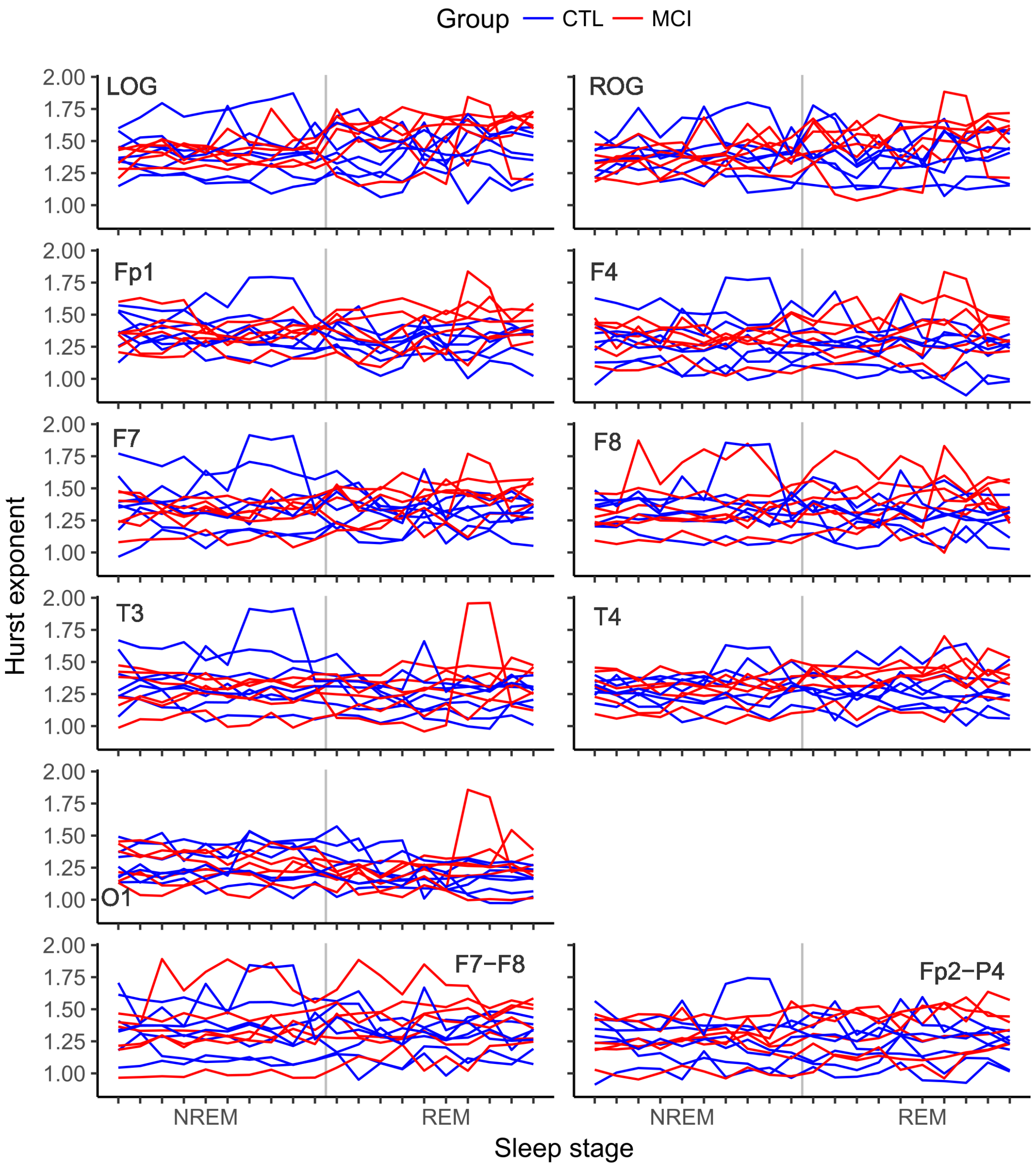

Figure S1: Hurst scaling values for each epoch at the transition from Non-Rapid Eye Movement (NREM) to REM sleep for both groups at the derivations where the ANOVA showed significant differences. CTRL, Control group (blue); Mild Cognitive Impairment group, MCI (red), (n = 13).

2.2 Tables

Table S1. Percentage of stationary epochs at full-night sleep by group and sleep stage and comparison between sleep stages (n = 13).

	CTL						MCI					
	NREM		REM		Wilcoxon test		NREM		REM		Wilcoxon test	
	Mean	SD	Mean	SD	W	p	Mean	SD	Mean	SD	W	p
Fp2	16.0	6.9	4.3	4.9	0.0	**0.016**	10.8	7.5	3.5	6.3	0.0	**0.031**
Fp1	17.0	8.3	5.8	9.2	1.0	**0.031**	13.3	11.2	3.7	7.2	0.0	**0.031**
F8	19.1	9.3	10.0	11.1	4.0	0.109	15.9	11.0	7.9	12.7	4.0	0.219
F7	20.2	5.9	7.6	6.0	0.0	**0.016**	15.1	10.5	4.5	9.7	0.0	**0.031**
F4	18.9	9.0	13.1	13.5	6.0	0.219	17.0	12.8	15.3	13.5	9.0	0.844
F3	19.1	6.5	9.8	9.6	2.0	**0.047**	17.1	13.3	15.6	20.9	6.0	0.438
T4	24.0	6.7	20.9	12.1	8.0	0.375	20.0	11.7	26.2	17.0	15.0	0.438
T3	29.0	6.0	26.8	11.1	10.0	0.578	22.2	12.1	23.5	18.7	13.0	0.688
C4	24.9	9.0	18.0	13.0	5.0	0.156	19.9	13.3	23.5	17.6	14.0	0.563
C3	24.6	5.2	19.2	11.1	7.0	0.297	19.9	11.2	30.5	21.0	19.0	0.094
T6	33.4	8.4	29.3	15.0	10.0	0.578	20.6	12.3	25.7	18.8	15.0	0.438
T5	34.6	15.1	33.8	18.1	13.0	0.938	26.6	17.1	32.4	16.0	15.0	0.438
P4	25.9	5.1	18.1	10.9	6.0	0.219	19.4	12.5	21.5	17.6	13.0	0.688
P3	26.6	8.1	22.1	11.1	7.0	0.297	22.4	12.9	26.9	17.5	14.0	0.563
O2	30.0	7.2	25.4	13.4	8.0	0.375	20.0	10.8	24.3	19.0	13.0	0.688
O1	32.8	11.9	32.8	17.6	15.0	0.938	21.7	14.6	27.2	19.0	15.0	0.438
FZ	21.1	7.0	16.2	13.1	7.0	0.297	19.1	13.7	19.7	16.6	12.0	0.844
CZ	20.3	7.4	14.3	9.6	7.0	0.297	18.0	11.4	16.6	11.8	10.0	1.000
PZ	22.3	4.4	16.5	9.1	5.0	0.156	20.5	15.4	22.7	15.9	13.0	0.688
LOG	43.4	12.8	16.6	13.0	0.0	**0.016**	36.3	9.4	10.0	8.7	0.0	**0.031**
ROG	46.2	16.2	22.2	16.4	0.0	**0.016**	35.5	15.6	14.6	11.9	0.0	**0.031**
EMG	27.6	28.5	31.2	29.4	25.0	0.078	12.6	15.7	14.2	14.9	11.0	1.000

CTRL, Control group; MCI, Mild Cognitive Impairment group.REM, Rapid Eye Movement sleep. NREM, Non-REM sleep. LOG, Left Oculogram. ROG, Right Oculogram. EMG, Electromyogram. Significant results for Wilcoxon tests are indicated in bold.

Table S2. Scaling results for each group in the transition from NREM to REM at individual derivations excluding one suspected outlier from each group (n = 11).

	CTRL		MCI		Mixed ANOVA					
	NREM	REM	NREM	REM	Group Df = 1, 22		Stage Df = 1, 22		Group×Stage Df = 1, 22	
	Mean (SD)	Mean (SD)	Mean (SD)	Mean (SD)	F	*p*	F	*p*	F	*p*
Fp2	1.30 (0.10)	1.28 (0.13)	1.34 (0.12)	1.45 (0.13)	2.68	0.136	4.06	0.075	12.33	**0.007**
Fp1	1.33 (0.10)	1.29 (0.12)	1.34 (0.12)	1.42 (0.13)	1.35	0.276	0.29	0.602	6.23	**0.034**
F8	1.30 (0.11)	1.28 (0.14)	1.34 (0.21)	1.42 (0.17)	1.14	0.314	1.19	0.304	4.95	**0.053**
F7	1.34 (0.19)	1.30 (0.13)	1.29 (0.12)	1.42 (0.12)	0.20	0.668	1.69	0.226	7.83	**0.021**
F4	1.27 (0.17)	1.23 (0.16)	1.29 (0.13)	1.36 (0.16)	0.71	0.422	0.27	0.617	8.51	**0.017**
F3	1.31 (0.19)	1.24 (0.14)	1.28 (0.13)	1.29 (0.12)	0.01	0.908	1.37	0.272	2.61	0.141
T4	1.28 (0.11)	1.27 (0.15)	1.27 (0.13)	1.35 (0.12)	0.20	0.668	2.07	0.184	3.54	0.092
T3	1.31 (0.16)	1.22 (0.13)	1.25 (0.13)	1.32 (0.12)	0.07	0.803	0.32	0.584	5.98	**0.037**
C4	1.27 (0.16)	1.22 (0.14)	1.27 (0.10)	1.29 (0.08)	0.25	0.626	0.43	0.526	2.00	0.191
C3	1.29 (0.15)	1.24 (0.15)	1.24 (0.13)	1.24 (0.10)	0.13	0.725	1.07	0.329	0.89	0.371
T6	1.17 (0.27)	1.09 (0.24)	1.28 (0.15)	1.32 (0.22)	1.50	0.252	0.60	0.459	3.46	0.096
T5	1.23 (0.11)	1.22 (0.15)	1.23 (0.13)	1.27 (0.16)	0.12	0.741	0.11	0.746	1.13	0.316
P4	1.24 (0.17)	1.17 (0.12)	1.24 (0.12)	1.26 (0.12)	0.31	0.590	1.47	0.256	2.37	0.158
P3	1.25 (0.16)	1.18 (0.12)	1.23 (0.11)	1.24 (0.11)	0.06	0.813	1.26	0.290	1.82	0.210
O2	1.29 (0.14)	1.19 (0.11)	1.26 (0.11)	1.25 (0.13)	0.06	0.815	2.42	0.154	1.80	0.212
O1	1.29 (0.13)	1.20 (0.13)	1.24 (0.12)	1.22 (0.11)	0.08	0.779	3.34	0.101	1.39	0.268
FZ	1.31 (0.16)	1.24 (0.15)	1.26 (0.13)	1.28 (0.11)	0.00	0.955	1.30	0.284	3.84	0.082
CZ	1.26 (0.13)	1.23 (0.17)	1.25 (0.12)	1.26 (0.12)	0.03	0.872	0.19	0.674	0.39	0.546
PZ	1.29 (0.23)	1.19 (0.13)	1.25 (0.11)	1.24 (0.09)	0.02	0.902	2.24	0.168	1.10	0.321
LOG	1.36 (0.13)	1.38 (0.16)	1.40 (0.10)	1.61 (0.11)	4.48	0.063	24.23	**0.001**	17.73	**0.002**
ROG	1.36 (0.12)	1.35 (0.16)	1.36 (0.12)	1.55 (0.11)	2.64	0.139	12.87	**0.006**	18.04	**0.002**
EMG	0.71 (0.40)	0.81 (0.30)	0.49 (0.12)	0.58 (0.09)	1.41	0.274	2.80	0.138	0.02	0.906

CTRL, Control group; MCI, Mild Cognitive Impairment group.REM, Rapid Eye Movement sleep. NREM, Non-REM sleep. LOG, Left Oculogram. ROG, Right Oculogram. EMG, Electromyogram. Significant results for ANOVA tests are indicated in bold. In each case, the Greenhouse-Geisser's correction for repeated measures was employed.
